# Extracellular vesicle-enriched secretome of adipose-derived stem cells upregulates clusterin to alleviate doxorubicin-induced apoptosis in cardiomyocytes

**DOI:** 10.1186/s13062-025-00664-5

**Published:** 2025-07-16

**Authors:** Wan-Tseng Hsu, Shinji Kobuchi, Tung-Chun Russell Chien, I-Chun Chen, Shohei Hamada, Masayuki Tsujimoto, I-Lin Tsai, Yun-Sheng Wong, Kuan-Hsuan Tung, Ying-Zhen He

**Affiliations:** 1 School of Pharmacy, College of Medicine, National Taiwan University, 33 Linsen S. Rd., R201, Zhongzheng Dist, Taipei, 100025 Taiwan; 2 Laboratory of Pharmacokinetics, Kyoto Pharmaceutical University, Kyoto, Japan; 3 Graduate Institute of Clinical Pharmacy, College of Medicine, National Taiwan University, Taipei, Taiwan; 4 Department of Medical Oncology, National Taiwan University Cancer Center, Taipei, Taiwan; 5 Department of Oncology, National Taiwan University Hospital, Taipei, Taiwan; 6 Graduate Institute of Oncology, College of Medicine, National Taiwan University, Taipei, Taiwan; 7 Division of Pharmaceutical Sciences, Kanazawa University, Kanazawa, Japan; 8 Laboratory of Clinical Pharmacy, Kyoto Pharmaceutical University, Kyoto, Japan; 9 Department of Biochemistry and Molecular Cell Biology, School of Medicine, College of Medicine, Taipei Medical University, Taipei, Taiwan

**Keywords:** Doxorubicin-induced cardiotoxicity, Adipose-derived stem cells, Extracellular vesicles, Secretome, Conditioned medium, Clusterin, Cardiomyocyte apoptosis

## Abstract

Doxorubicin (DOX) is a potent chemotherapeutic widely used against various cancers, but its clinical application is limited by DOX-induced cardiotoxicity (DIC). This study explored the cardioprotective potential of extracellular vesicle-enriched secretome derived from adipose stem cells (EVS_ASC_) in mitigating DOX-induced apoptosis in cardiomyocytes. Adipose-derived stem cells were cultured, and their conditioned medium and extraceullular vesicles were isolated and characterized according to the Minimal Information for Studies of Extracellular Vesicles 2023 guidelines. HL-1 cardiomyocytes were pretreated with EVS_ASC_ before exposure to 1 µM DOX. Cell viability was assessed via the cell counting kit-8 assay, while apoptosis markers and survival mediators were evaluated through Western blotting. RNA sequencing identified differentially expressed genes, including clusterin (*Clu*), which was further quantified using an enzyme-linked immunosorbent assay. The functional role of clusterin was validated through siRNA-mediated knockdown. EVS_ASC_ significantly improved cell viability in DOX-exposed cardiomyocytes and reduced the cleaved caspase-3 to procaspase-3 ratio. Clusterin expression was highest in EVS_ASC_-treated cells, and its knockdown markedly increased caspase-3 cleavage, confirming its pivotal role in cardioprotection. Moreover, EVS_ASC_ enhanced the phosphorylation of AKT, Bcl2-associated agonist of cell death, and glycogen synthase kinase-3β, implicating PI3K/AKT pathway activation in clusterin upregulation and anti-apoptotic effects. These findings demonstrate that EVS_ASC_ mitigates DOX-induced apoptosis in cardiomyocytes through clusterin upregulation and PI3K/AKT pathway activation. Clusterin is identified as a potential biomarker for evaluating EVS_ASC_ efficacy. While EVS_ASC_ shows promise as a cardioprotective strategy against DIC, further studies are needed to optimize its therapeutic safety by addressing potential oncogenic risks.

**Supplementary Information:**

The online version contains supplementary material available at 10.1186/s13062-025-00664-5.

## Introduction

Cancer, a major global health challenge, is a leading cause of morbidity and mortality worldwide, with millions of new cases being diagnosed every year [1]. Among the available chemotherapeutic agents, doxorubicin (DOX) has proven effective against multiple cancers, including breast cancer and Hodgkin’s lymphoma [2]. DOX exerts its potent anticancer effects primarily by intercalating DNA, inhibiting topoisomerase-II activity, and increasing reactive oxygen species (ROS) levels [3, 4]. However, the clinical use of DOX is limited by its potential severe cardiac side effects, known as DOX-induced cardiotoxicity (DIC) [5–9]. These side effects undermine the therapeutic benefits of DOX. Thus, the anticancer efficacy of DOX must be balanced with the risk of DIC. Addressing DIC, which is a key focus in current oncological research, may help expand the clinical application of DOX, and despite its side effects, DOX remains a cornerstone in cancer treatment.

A complex interplay of several mechanisms mediates the pathogenesis of DIC. These mechanisms include interference with nucleic acid and protein synthesis, increased accumulation of cytosolic iron, dysregulation of intracellular calcium signaling, and bioenergetic collapse of the mitochondria [10, 11]. Notably, the role of apoptosis in DIC was previously underestimated. However, apoptosis is now recognized as a crucial factor contributing to DIC [12]. Oxidative stress, which particularly threatens cardiomyocytes because of their inherently low antioxidative capacity, plays a central role in DIC-related apoptosis [13–15]. Consequently, current research on DIC is focused on developing preventive and therapeutic interventions targeting oxidative stress, inflammation, cell death, and other mechanisms [15, 16].

DIC involves both the intrinsic and extrinsic apoptotic pathways, which are tightly interconnected [17, 18]. The intrinsic (mitochondrial) pathway is of particular importance [19]. It is strictly regulated by the B-cell lymphoma (Bcl)-2 family of proteins, which modulate the balance between proapoptotic and antiapoptotic signals [18–20]. DOX induces intrinsic apoptosis primarily by inhibiting the phosphatidylinositol 3-kinase (PI3K)/protein kinase B (AKT) pathway, a major regulator of cell survival [21]. Inhibition of AKT activity leads to the dephosphorylation of the proapoptotic protein Bcl-2-associated death (BAD), enabling it to bind antiapoptotic proteins such as Bcl-2 and Bcl-xL and thus neutralize their protective effects. This interaction facilitates the release of cytochrome c from the mitochondria and activates downstream caspases, culminating in apoptosis [21–23]. In parallel, DOX activates the tumor suppressor protein p53, which facilitates apoptosis by enhancing caspase-3 activation through the extrinsic pathway [18, 24]. The convergence of these pathways on caspase activation demonstrates the multifaceted and tightly regulated nature of DOX-induced apoptosis in cardiomyocytes.

Currently, dexrazoxane (DRZ) is the only drug that has been approved by the US Food and Drug Administration for the prevention of DIC. The American Society of Clinical Oncology recommends its use alongside high-dose anthracyclines [25]. However, a 2024 study suggested that DRZ can cause secondary malignancies and worsen chemotherapy-mediated myelosuppression; these concerns limit the overall therapeutic benefits of DRZ [26] and have driven ongoing research into effective DIC therapies [27].

Advances have been made in stem cell therapy for cardiac regeneration. Mesenchymal stem cells (MSCs) have gained particular attention because of their paracrine effects, low immunogenicity, and multipotency [28–30]. MSCs are well known for their substantial extracellular vesicle (EV) production [31]. Notably, MSCs can be isolated from several sources, such as adipose tissue (adipose-derived stem cells [ASCs]), bone marrow (bone marrow–derived MSCs [BM-MSCs]), and umbilical cords (umbilical cord–derived MSCs [UC-MSCs]), with each source offering distinct advantages [32]. ASCs are widely used in stem cell therapy because of their abundance, ease of harvest, regenerative potential, and minimal risks [33, 34]. These cells exhibit resistance to early senescence and can mitigate inflammation, apoptosis, and oxidative stress, thereby preventing DIC [35]. Thus, ASCs have promising applications in therapeutic strategies targeting DOX-induced apoptosis.

Lai et al. demonstrated the efficacy of MSC-derived EVs in treating ischemic heart disease [36]. Subsequent research investigated the cardioprotective potential of EVs [37], demonstrating that they can enhance cell survival and function [36, 38–40]. EVs, including exosomes and microvesicles, are key components of the ASC-derived secretome [41]. These vesicles carry a diverse range of proteins, lipids, and nucleic acids that influence the biological process and function of recipient cells [42, 43]. This characteristic renders EVs a promising therapeutic strategy for cardiovascular conditions, particularly DIC [44]. Although the therapeutic effects of EVs derived from BM-MSCs [45] and UC-MSCs [46] have been extensively studied, the therapeutic potential of ASC-derived EVs for DIC remains underexplored. Moreover, studies on this topic have generally focused on combined DOX- and trastuzumab-induced cardiotoxicity [47] and DOX-induced senescence [48]. Thus, whether ASC-derived EVs can prevent DOX-induced apoptosis remains unclear, and the effects of EV-enriched secretome derived from ASCs (EVS_ASC_) on DOX-induced apoptosis warrant further research. Additionally, studies should be conducted to identify the specific effector molecules responsible for the therapeutic effects of EVS_ASC_ on cardiomyocytes, given that a lack of such information has hindered the development of EVS_ASC_ as a treatment option for cardiomyocyte injury.

In this study, we evaluated the efficacy of EVS_ASC_ in mitigating DIC. We hypothesized that EVS_ASC_ would reduce DOX-induced oxidative stress and cardiomyocyte apoptosis. Our results suggest that EVS_ASC_ attenuates DOX-induced mitochondrial dysfunction and apoptosis in cardiomyocytes. By elucidating the mechanisms underlying the therapeutic effects of EVS_ASC_ and directly comparing its efficacy with that of the established cardioprotective agent DRZ, we highlight EVS_ASC_ as a promising cardioprotective strategy for patients receiving DOX-based chemotherapy.

## Materials and methods

### Isolation and culture of ASCs

Female FVB/N mice (age: 12 weeks; weight: 20 ± 2 g; *n* = 17) were obtained from the National Institutes of Applied Research National Center for Biomodels, Taipei, Taiwan. In this study, we selected female FVB/N mice for ASC isolation to ensure consistency with our long-term research goals involving breast cancer–bearing mouse models. FVB/N mice have been widely used in cardiovascular and oncologic research [49, 50] because of their well-characterized cardiac responses, stable metabolic profiles, and compatibility with the mammary-specific polyomavirus middle T antigen overexpression (MMTV-PyMT) transgenic model of mammary tumorigenesis [51]. Using female mice enabled us to generate ASCs that are genetically and physiologically aligned with the goals of our future studies, which will evaluate the potential of ASC-derived EVs for both cardioprotection and tumor modulation within an immunocompetent system.

The mice were housed under standard conditions at the Laboratory Animal Center of the National Taiwan University College of Medicine. All animal experiment protocols were approved by the Institutional Animal Care and Use Committee (approval number: 20201104). Euthanasia was performed by placing the mice in a clear, sealed acrylic chamber and releasing carbon dioxide (CO_2_) from high-pressure cylinders. The flow rate was controlled using a gas control valve, with maintenance of a volume replacement rate of 30–70% per minute and a target rate of 40% per minute to ensure humane and gradual euthanasia.

After euthanasia, ASCs were isolated from the inguinal white adipose tissue and the subcutaneous fat depot extending from the dorsal pelvis to the thigh of the hind limb. These anatomical sites were selected to ensure consistency in sample collection. The cells were isolated following a previously described protocol [52]. All procedures were performed following the Animals in Research: Reporting In Vivo Experiments guidelines 2.0.

The isolated ASCs were cultured in MesenPro media (Gibco, USA) and optimized for MSC expansion. Primary ASCs were trypsinized and passaged to promote further expansion. For the experiments, ASCs between passages 2 and 7 were used to ensure consistency in cell viability. First, differentiation assays were performed to evaluate adipogenic and osteogenic potential and to analyze the phenotypic profile of the ASCs [52]. After, flow cytometry was performed to assess the expression of specific surface markers, such as CD44, CD105, CD90, CD11b, and CD34. All antibodies used in flow cytometry, along with the appropriate isotype controls, were obtained from BioLegend (USA).

### Collection of conditioned medium from ASCs

To standardize the secretory profile and eliminate the confounding effects of cell proliferation and overconfluence, the ASCs were pretreated with mitomycin C (25 µg/mL; Sigma-Aldrich, USA) for 30 min, which induced irreversible cell cycle arrest. This strategy was adopted to ensure that the collected conditioned medium (CM) reflected the paracrine output of a metabolically active but nondividing ASC population, enhancing experimental consistency and reducing batch-to-batch variability due to differences in cell density, growth phase, or proliferation-related changes.

Mitomycin C-treated ASCs were seeded into 12-well plates in MesenPro media at a density of 1.1 × 10^5^ cells per well (equivalent to 3.1 × 10^4^ cells/cm^2^). After 24-h incubation to allow for cell attachment and stabilization, the culture medium was replaced with Dulbecco’s Modified Eagle Medium-High Glucose (DMEM-HG; Hyclone, USA) supplemented with 10% fetal bovine serum (FBS; Merck, USA). Subsequently, the cells were incubated for an additional 72 h to allow for the accumulation of secreted bioactive factors in the medium. After incubation, the CM was harvested and centrifuged at 1000 ×*g* for 10 min at 4°C to remove cellular debris. Next, the supernatant was passed through a 0.22-µm filter to ensure sterility and eliminate residual particles. The final CM had a protein concentration of 2.78 ± 0.12 mg/mL. It was aliquoted and stored at − 80 °C until further use in downstream experiments.

### Establishment of an in vitro DIC model

Mouse HL-1 cardiomyocytes (Sigma-Aldrich) were cultured in Complete Claycomb medium consisting of Claycomb medium (Sigma-Aldrich) supplemented with 100 µM norepinephrine (Sigma-Aldrich), 10% FBS (Merck), 1% (v/v) penicillin/streptomycin/amphotericin B (Corning, USA), and 4 mM L-glutamine (Biological Industries, Israel). HL-1 cells between passages 7 and 10 were used in all experiments.

To ensure optimal cell adhesion, viability, and contractile function, culture plates were precoated with 0.02% gelatin and 5 µg/mL fibronectin in accordance with standard Claycomb protocols. DOX hydrochloride (Selleckchem, USA) was first dissolved in dimethyl sulfoxide to prepare a 10 mM stock solution, which was subsequently diluted in DMEM-HG to prepare final working concentrations of 0.1, 0.5, 1, and 2 µM. Each solution was thoroughly vortexed to ensure complete dissolution. HL-1 cardiomyocytes were then treated with DOX-containing medium and incubated at 37 °C for 24 h to induce cardiotoxic injury.

To enable assessment of the therapeutic effects of EVS_ASC_, HL-1 cardiomyocytes were treated with 50 µM DRZ (Cayman, USA), CM, or EVs for 4 h in 10% FBS-supplemented DMEM-HG before DOX exposure. The culture media were replenished and maintained throughout DOX exposure. EVs were administered at a dose of 2 × 10^4^ particles per HL-1 cell. The volume of CM used was scaled according to the culture plate format: 100 µL for 96-well plates, 1 mL for 12-well plates, and 2 mL for 6-well plates. After incubation for 24 h, the DOX-containing medium was removed, and the cells were washed once with phosphate-buffered saline (PBS) to remove the residual drug. Subsequently, cell viability was assessed using the cell counting kit-8 (CCK-8) assay, following the manufacturer’s instructions.

Human induced pluripotent stem cell (hiPSC)-derived cardiomyocytes were cultured following the manufacturer’s instructions (Cell Applications, USA). In brief, hiPSC-derived cardiomyocytes were thawed in a 37 °C water bath, gently resuspended in i-HCm Plating Medium (Cell Applications), and plated (density: 2 × 10^5^ cells/cm^2^) onto 96-well plates precoated with the i-HCm coating solution (Cell Applications). The cardiomyocytes were maintained at 37 °C in a 5% CO_2_ humidified incubator for 8 days, during which time the cells spontaneously initiated rhythmic contractions. Subsequently, the cells were treated with 1 µM DOX for 24 h to induce cardiotoxic stress. Spontaneous beating activity of the cells was recorded both before and after treatment to assess contractile function. Recordings were obtained using an inverted phase-contrast microscope (Olympus CKX41; GoYoung, Taiwan) equipped with a high-resolution digital camera (EOS800D; Canon, Taiwan) mounted onto the microscope. For each experimental condition, videos were recorded at 20× magnification for a duration of 10 s per well, capturing at least three independent fields per well to account for variability in beating patterns. The videos were saved in the MP4 format for subsequent analysis. The beating rate (beats per minute) was manually calculated by counting the number of contractions observed during the 10-s videos, and values were normalized to beats per minute. Two independent, blinded investigators performed all analyses to ensure accuracy and minimize bias.

### Transwell coculture of ASCs and HL-1 cardiomyocytes

To investigate the paracrine effect of ASCs on HL-1 cardiomyocytes in the absence of direct cell contact, we used a 24-mm Transwell system (Corning, USA) equipped with a 0.4-µm pore polyester membrane insert. In brief, ASCs were seeded into the upper chamber of the Transwell system at a density of 2 × 10^5^ cells per insert (4.67 cm^2^) in 1.5 mL of Complete Claycomb medium, which contained 10% FBS, 0.1 mM norepinephrine, 2 mM L-glutamine, and 1% penicillin/streptomycin. Simultaneously, HL-1 cardiomyocytes were seeded into the lower chambers of a 6-well plate at the same density (2 × 10^5^ cells per well) in the same Complete Claycomb medium to ensure shared culture conditions. Notably, the ASCs were not prewashed with PBS before coculture, considering that both cell types were exposed to the same FBS-containing medium throughout the experiment.

The cells were cocultured for 4 h to allow for cellular adaptation and paracrine signaling. After, HL-1 cardiomyocytes in the lower chamber were treated with DOX to induce DIC. During DIC induction, the ASCs remained in the upper chamber and were exposed to DOX through diffusion within the shared medium. This setup preserved continuous paracrine interactions. In addition, it facilitated the assessment of ASC-mediated protective effects under coexposure conditions.

### CCK-8 assay

To evaluate the metabolic activity of living cells—an indicator of posttreatment cell viability, CCK-8 assays were performed following the manufacturer’s instructions. After relevant pretreatment (DRZ, CM, or EVs) and DIC induction, HL-1 cardiomyocytes were washed with PBS. Subsequently, the cells were exposed to a CCK-8 solution (Dojindo, Japan) diluted to 10% (v/v) in Complete Claycomb medium and incubated for 4 h at 37 °C. After incubation, absorbance was measured at 450 nm by using a microplate reader. Notably, absorbance is directly correlated with the number of viable cells. Each experiment was conducted at least in triplicate. The metabolic activity of living cells is expressed as a percentage of that noted in untreated cells.

### Annexin V–propidium iodide assay

Apoptosis in HL-1 cardiomyocytes was assessed using the Apoptosis (Annexin V) Plate Assay Kit (Dojindo, Japan), following the manufacturer’s instructions. This kit contains fluorescein isothiocyanate–labeled annexin V. In brief, the cells were seeded (density: 2.5 × 10^5^ cells per well) into 12-well plates and were cultured in Complete Claycomb medium for 24 h. After respective treatment, the cells were centrifuged at 400 ×*g* for 5 min at 4 °C, washed twice with PBS containing 2% FBS, and resuspended in annexin V binding solution at a density of 1 × 10^6^ cells/mL. A 100-µL aliquot of the cell suspension was transferred to a microcentrifuge tube, and 3 µL of propidium iodide (PI) and 5 µL of annexin V were added to the cell suspension and incubated at room temperature for 15 min. After incubation, 400 µL of annexin V binding solution was added to the sample. Flow cytometry was performed using the BD FACSLyric instrument and BD FACSuite software (BD Biosciences, USA). Annexin V-positive and PI-negative cells were regarded as early apoptotic cells.

### Quantification of mitochondrial superoxide production through high-content imaging and flow cytometry

Mitochondrial superoxide production was assessed using a costaining method with several fluorescent dyes: Hoechst 33342 (blue; Dojindo) for nuclear staining, MitoBright LT Green (green; Dojindo) for mitochondrial mass staining, and mtSOX Deep Red (purple; Dojindo) for mitochondrial superoxide staining. High-content imaging (HCI) of HL-1 cardiomyocytes was performed using the ImageXpress Micro Confocal system (Molecular Devices, USA), which enabled high-resolution visualization and quantitative analysis of fluorescence intensity. HCI provided a detailed assessment of mitochondrial superoxide levels in HL-1 cardiomyocytes. The captured images were analyzed to quantify the production of mitochondrial ROS. Furthermore, the cells were resuspended in a medium supplemented with mtSOX Deep Red (5 µM) and incubated for 30 min at 4 °C. Flow cytometry was performed using a BD FACSLyric instrument. Thus, mitochondrial superoxide production was precisely evaluated under various experimental conditions.

### Seahorse XF mitochondrial stress assay

To enable measurement of the oxygen consumption rate (OCR) of HL-1 cardiomyocytes, the seahorse XF mitochondrial stress assay was performed using the Seahorse XFe96 Analyzer (Agilent, Santa Clara, USA). The cells were seeded (density: 1.2 × 10^4^ cells per well) into Cell-Tak-coated 96-well Seahorse plates and incubated overnight in a CO_2_ incubator at 37 °C. The next day, the cells were subjected to relevant pretreatment and treatment. After treatment, the medium was carefully removed, and 180 µL of Seahorse assay medium (sodium bicarbonate–free Dulbecco’s Modified Eagle Medium supplemented with glucose and glutamine) was added to each well. The plates were incubated in a CO_2_-free incubator at 37 °C for 1 h to equilibrate the cells before the assay. OCRs were measured under basal conditions and following sequential additions of various compounds, namely, 1 µM oligomycin (added to inhibit ATP synthase and measure proton leak respiration), 1 µM carbonyl cyanide-4-(trifluoromethoxy) phenylhydrazone (added to uncouple oxidative phosphorylation and determine maximal respiration), and 0.5 µM rotenone plus antimycin A (added to inhibit complexes I and III, respectively, and calculate nonmitochondrial respiration). Notably, the plates were incubated for 30 min at 37 °C in the absence of CO_2_ before OCR measurement. Data were analyzed using the Wave software (Agilent) to evaluate mitochondrial function, including basal respiration and maximal respiration.

### Qualitative and quantitative analyses of proteins with cardioprotective potential

The Milliplex kit (Merck) was used to quantitatively analyze proteins with cardioprotective potential. This kit enabled the simultaneous analysis of several well-known proteins with cardioprotective potential: insulin-like growth factor, amphiregulin, placental growth factor-2, epidermal growth factor, β-fibroblast growth factor, interleukin-6, prolactin, vascular endothelial growth factor-A, and hepatocyte growth factor. Mouse Cytokine Array Q4000 (RayBio, USA) and Growth Factor Cytokine Antibody Arrays (RayBio) were used to comprehensively assess the cytokines, chemokines, and growth factors present in the CM or EVs collected from a 72-h culture of ASCs. This combination facilitated detailed profiling of the key factors contributing to the cardioprotective potential of EVS_ASC_.

### Isolation of EVs from ASCs

EVs were purified from the CM of ASCs through size exclusion chromatography [53, 54]. In brief, the CM was collected from ASCs (from passages 5 to 7) cultured for 72 h in a medium supplemented with exosome-depleted FBS (Gibco, USA) at 37 °C in a 5% CO_2_ humidified incubator. The CM was first centrifuged and then filtered through a 0.22-µm filter to remove larger particles, cells, and debris. The resulting sample was further concentrated and purified using the qEV Isolation technique with a qEV1 (35 nm) column (Izon Science, New Zealand), thereby enabling preparation of a highly purified sample of intact vesicles. The protein concentration of the purified EVs was determined using the bicinchoninic acid assay (iNtRON Biotechnology, Korea). The final preparation had a protein concentration of 0.3 µg/µL, indicating batch consistency. The total protein concentration in each EV preparation was quantified. The samples were stored at − 80 °C for use in future experiments.

### Measurement of particle size, concentration, and zeta potential of EVs with tunable resistive pulse sensing

The particle size, concentration, and zeta potential of EVs were measured using the tunable resistive pulse sensing technology [54, 55] with the IZON Exoid instrument (Izon Science). To measure size and concentration, the NP200 nanopore (serial number: C00285) was used and calibrated with CPC100 particles (batch ID: 20230718; mean diameter: 95 nm; Izon Science). The measurements were performed using the IZON ECS software (version v1.4.0.88) at a voltage of 1600 mV and a stretch of 48.99 mm. Zeta potential was measured using the NP100 nanopore (serial number: C01560) with identical CPC100 calibration particles. The measurements were performed at a voltage of 750 mV and a stretch of 48.99 mm. This approach ensured precise characterization of EV properties, enabling robust analysis of size distribution, concentration, and surface charge.

### Characterization of EVs

To determine the presence of the surface marker CD63 on EVs, we characterized the vesicles through flow cytometry (AMNIS ImageStreamX Mark II Flow Cytometer; AMNIS/Millipore, USA) [56]. Western blotting was performed to detect the expression of internal markers such as TSG101, ALG-2 interacting protein X (ALIX), and calnexin. The following primary antibodies were used in Western blotting: anti-ALIX (dilution: 1:1000; catalog number: 12422-1-AP; Proteintech, USA), anti-TSG101 (dilution: 1:500; catalog number: 28283-1-AP Proteintech), anti-calnexin (dilution: 1:1000; catalog number: 66903-1-Ig; Proteintech), and anti-CD63 (10 mg/L; catalog number: 143920; BioLegend).

### Transmission electron microscopy

After purification with the qEV column, a 10-µL droplet of each EV sample in PBS was placed onto a grid and incubated for 7 min at room temperature to facilitate particle adherence to the grid. After, the grid was immersed in a 10-µL droplet of 1.75% uranyl acetate (w/v) for negative staining. Subsequently, it was analyzed under a transmission electron microscope (Hitachi-7650; Hitachi Science & Technology, UK).

### Assessment of EV uptake by HL-1 cardiomyocytes

EVs were labeled using the Exosparkler Exosome Membrane Labeling Kit (Dojindo, Japan), following the manufacturer’s instructions. HL-1 cardiomyocytes were seeded (density: 2 × 10^4^ cells/cm^2^) into 96-well culture plates. Labeled EVs were added to the plates (100 µL per well) and incubated at 37 °C for 4 h. After incubation, free EVs were removed by washing thrice with PBS. EV uptake was visualized at specific time points through live cell confocal microscopy, which was performed using the ImageXpress Micro Confocal High-Content Imaging System (Molecular Devices).

### RNA sequencing and differential gene expression analysis

Total RNA was extracted from HL-1 cardiomyocytes using TRIzol (Invitrogen, USA). The quantity of purified RNA was measured using an ND-1000 spectrophotometer (Nanodrop Technology, USA). Absorbance was measured at 260 nm. The quality of the obtained RNA was assessed using a Bioanalyzer 2100 system (Agilent Technologies, USA) and an RNA 6000 LabChip kit (Agilent Technologies). RNA libraries were constructed using the SureSelect Strand-Specific RNA Library Preparation Kit (Agilent Technologies) and were size-selected using AMPure XP beads (Beckman Coulter, USA). Subsequently, RNA sequencing (RNA-seq) was performed using Illumina’s sequencing-by-synthesis technology (Illumina, USA). The resulting FASTQ reads were processed using Welgene Biotech’s (Taiwan) pipeline. The bcl2fastq software (version 2.20; Illumina, USA) was used for base-calling. Adapter sequences were removed, and sequence quality was improved through data trimming with Trimmomatic (version 0.36), for which a sliding-window approach was adopted. The reference genome used was GRCm38. The alignment was conducted using the Hierarchical Indexing for Spliced Alignment of Transcripts (HISAT) tool.

The expression level of each transcript was calculated from raw data by using the transcripts per million (TPM) normalization method; this method facilitated intersample comparisons [57]. Differentially expressed genes (DEGs) were analyzed using StringTie (version 2.1.4) and DEseq (version 1.39.0) or DEseq2 (version 1.28.1). Genome bias detection and correction were performed using Welgene Biotech’s proprietary pipeline. Functional enrichment analysis of DEGs was performed using the clusterProfiler software (version 3.6). Genes with a *p* value of ≤ 0.05 and a ≥ 2-fold change in expression level were considered to exhibit significantly different expression patterns. To generate heat maps, transcripts with an average TPM of > 5.0 across all samples were subjected to hierarchical clustering.

### Western blotting

HL-1 cardiomyocytes were washed as follows: the culture medium was carefully discarded from the dishes, and the cells were gently washed twice with ice-cold PBS to remove any residual medium and serum proteins. After washing, the PBS was aspirated completely, and ice-cold MILLIPLEX MAP Lysis Buffer (catalog number: 43 − 040; Merck Millipore) supplemented with protease/phosphatase inhibitor cocktail (catalog number: 5872 S; Cell Signaling Technology, USA) was added directly to the cells on ice. After, the cells were scraped using a prechilled plastic cell scraper, and the lysates were collected into prechilled microcentrifuge tubes. The tubes were gently agitated for 1 h at 4 °C to facilitate thorough protein extraction. Afterward, the samples were centrifuged at 14,000 ×*g* for 15 min at 4 °C. The supernatants, containing the soluble protein fractions, were carefully transferred into fresh, prechilled tubes and kept on ice for immediate use or stored at − 80 °C for future use. The insoluble pellets were discarded.

The protein concentration of each sample was determined using the bicinchoninic acid assay (iNtRON Biotechnology). Equal amounts of protein from each sample were separated through sodium dodecyl sulfate polyacrylamide gel electrophoresis. The resulting protein bands were then transferred onto polyvinylidene fluoride membranes (Immobilon-PSQ; Merck Millipore, USA). The membranes were blocked with protein-free blocking buffer (BIO-DOC, Taiwan) for 1 h. After washing, the membranes were incubated overnight at 4 °C with primary antibodies against the following proteins: caspase-3, cleaved caspase-3, AKT, phosphorylated AKT (Ser473), phosphorylated AKT (Thr308), BAD, phosphorylated BAD (Ser 112), glycogen synthase kinase (GSK)-3β, and phosphorylated GSK-3β (Ser9). All antibodies were obtained from Cell Signaling Technology. After incubation, the blots were washed thrice with 1X Tris-buffered saline (Omics Bio, Taiwan) containing 1% Tween 20 (Sigma-Aldrich); each wash lasted 10 min. Next, the membranes were incubated with horseradish peroxidase–conjugated secondary antibodies for 1 h at room temperature. Immunoreactive proteins were detected using enhanced chemiluminescence reagent kits (PerkinElmer, USA).

### Enzyme-linked immunosorbent assay (ELISA)

The concentrations of clusterin in both the cell culture supernatants and cell lysates were measured using enzyme-linked immunosorbent assay kits (R&D Systems, USA). The detection limit was set at 0.8 ng/mL.

### Small interfering RNA transfection for gene knockdown

HL-1 cardiomyocytes were seeded (density: 5 × 10^5^ cells per well) into 6-well plates and incubated for 24 h in a complete medium. Subsequently, the cells were transfected with relevant siRNAs (Sigma-Aldrich) by using the Lipofectamine RNAiMAX Transfection Reagent (Invitrogen). After 48 h, the transfected cells were pretreated with the CM or EVs for 4 h and then treated with DOX for 24 h. Cell lysates were obtained and analyzed through immunoblotting. The following siRNAs used were for gene knockdown: siCLU SASI Mm01_00065960 (sense strand: GUUCUUCGCCCGUGAGCUU [dT][dT]; antisense strand: AAGCUCACGGGCGAAGAAC [dT][dT]) and MISSION siRNA Universal Negative Control #1 (sense strand: GAUCAUACGUGCGAUCAGA[dT][dT]; antisense strand: UCUGAUCGCACGUAUGAUC[dT][dT]).

### PI3K/AKT Inhibition assay

To investigate the role of the PI3K/AKT pathway in CM-mediated cardioprotection, HL-1 cardiomyocytes were treated with the selective PI3K inhibitor LY294002 (Selleckchem). LY294002 was dissolved in dimethyl sulfoxide to prepare a 10 mM stock solution, which was diluted in culture medium to prepare final working concentrations of 5, 10, and 20 µM immediately before use. Control experiments revealed that treatment with 5–20 µM LY294002 alone did not induce significant cytotoxicity or apoptosis under the experimental conditions used. HL-1 cells were pretreated with LY294002 (20 µM) for 2 h before 24-h incubation with DOX (1 µM) in the presence or absence of CM. Subsequently, caspase-3 cleavage was assessed through Western blotting to evaluate the level of apoptosis. The experimental groups were as follows: control (no treatment), DOX-only (treatment with only DOX), DOX + CM (treatment with both DOX and CM), DOX + CM + LY294002 (treatment with DOX, CM, and LY294002), and reagent control (treatment with only LY294002).

### Statistical analysis

All data are presented as mean ± standard deviation values. Statistical analyses were performed using Prism (version 10; GraphPad Software, USA). Data normality was assessed using the Shapiro–Wilk test. On the basis of the data distribution, either the parametric or the nonparametric test was performed. One-way analysis of variance was used for intergroup comparisons. In the case of significant results (i.e., a significant F-statistic value), post hoc pairwise comparisons were performed using an independent *t* test. To mitigate the risk of type I error due to multiple comparisons, Bonferroni correction was performed, with adjustment of the resulting *p* values, and a corrected *p* value of < 0.05 was considered significant. The limited number of predefined comparisons in our study design justified the use of Bonferroni correction.

## Results

### DRZ modulates the concentration-dependent cytotoxicity of DOX in HL-1 cardiomyocytes

**Fig. 1:**
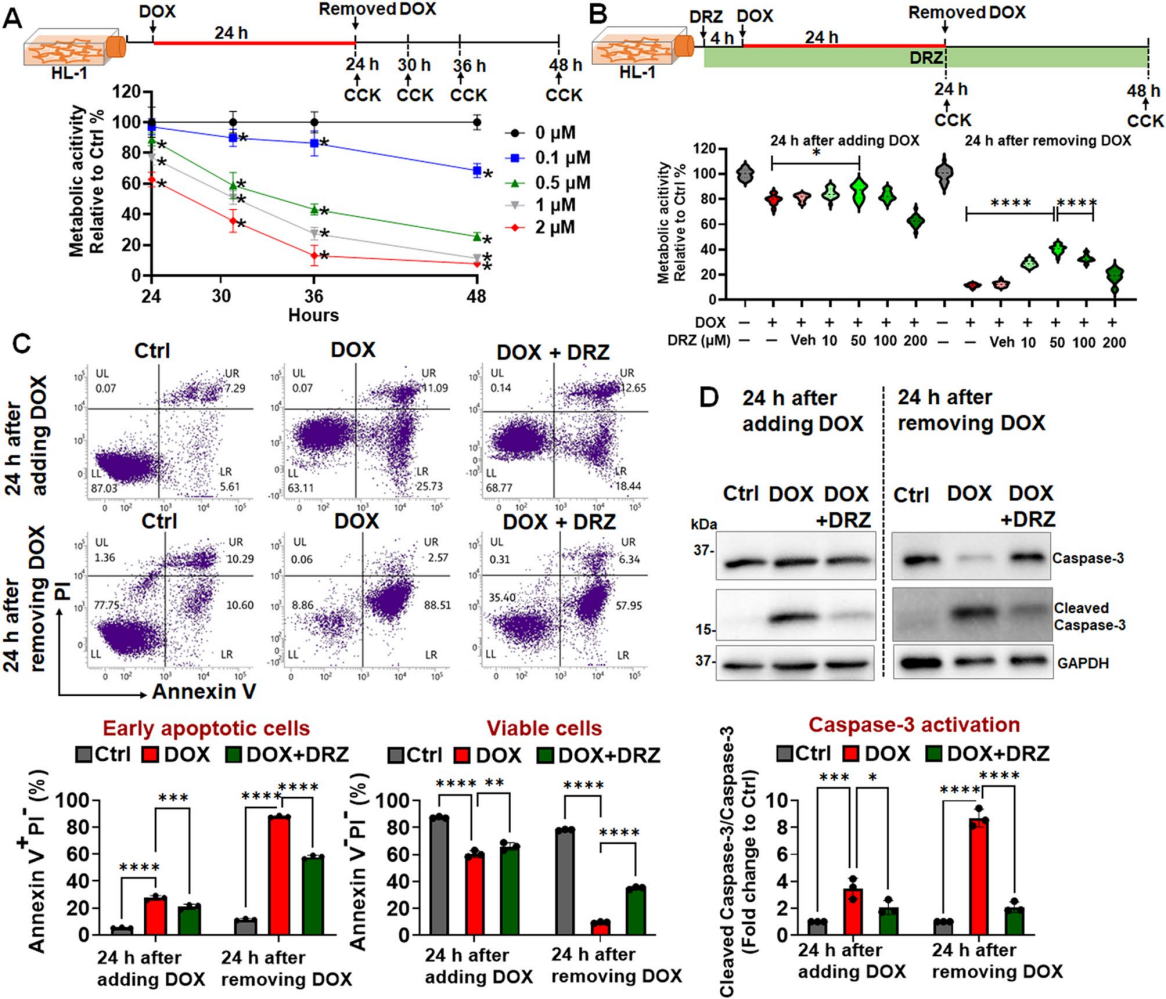
Persistent doxorubicin-induced cardiomyocyte damage and apoptosis despite dexrazoxane interventions **(A)** Cardiomyocytes were treated with 1 µM doxorubicin (DOX) for 24 h and subsequently cultured in a fresh medium without DOX for an additional 24 h. Cell viability was assessed using a CCK-8 assay. The results demonstrate a significant reduction in cell viability following DOX treatment (*p < 0.05 vs. 0 µM), which persists even after the removal of the drug, indicating irreversible viability loss. **(B)** Treatment with dexrazoxane (DRZ) at 50 µM restored cell viability to levels comparable to control, whereas concentrations above 100 µM appeared to have diminished protective effects. **(C)** Apoptotic cells were detected using annexin V staining. Representative images show increased annexin V-positive cardiomyocytes after 24 h of DOX treatment and continued apoptosis 24 h post-drug removal. Quantitative analysis of annexin V-positive cells confirmed significant apoptosis induced by DOX, which remains elevated after the removal of the drug. **(D)** Western blotting of cleaved caspase-3 in cardiomyocyte lysates. Cropped gels and blots are displayed, with full-length blots provided in Supplementary Fig. 1. The results demonstrate increased levels of cleaved caspase-3 following DOX treatment, which remain elevated even after drug removal, suggesting persistent activation of the apoptotic pathway. *p < 0.05; **p < 0.01; ***p < 0.005; ****p < 0.0001

Treatment with DOX at doses of 15–90 mg/m^2^ typically results in steady-state plasma concentrations of 25–250 nM [58]. To investigate the irreversible effects of DOX on the metabolic activity of cardiomyocytes, we treated HL-1 cells with DOX concentrations ranging from 0.1 to 2 µM (Fig. 1A). Over a 48-h period, treatment with 0.1 µM DOX gradually reduced the metabolic activity of the HL-1 cells to approximately 80% of that noted in control cells (Fig. 1A). The effect of DOX became stronger at higher concentrations (0.5, 1, and 2 µM; Fig. 1A). Specifically, metabolic activity decreased to approximately 30% in cells treated with 0.5 µM DOX and to ≤ 20% in those treated with 1 or 2 µM DOX (Fig. 1A).

We subsequently evaluated the protective effects of DRZ pretreatment on HL-1 cells treated with 50 µM DOX. Pretreatment with DRZ restored the metabolic activity of the DOX-treated (24 h) cells to approximately 86% of that noted in control cells. This indicates that DRZ can significantly reduce the extent of DOX-induced DIC (Fig. 1B). However, the protective efficacy of DRZ decreased at concentrations beyond 100 µM. Furthermore, the results revealed no significant increase in metabolic activity with 100 µM DRZ compared with the activity observed with 50 µM DRZ, indicating a potential upper limit to the protective benefits of DRZ. Overall, these findings indicate that higher concentrations of DRZ do not confer additional protection and potentially even exert adverse effects (Fig. 1B).

### DRZ partially mitigates DOX-induced apoptosis in HL-1 cardiomyocytes

We investigated whether 1 µM DOX induces apoptosis in HL-1 cardiomyocytes. This concentration was selected because it has been demonstrated to induce observable cardiotoxicity in various cellular models, including HL-1 cells [59] and hiPSC-cardiomyocytes [60], and to yield consistent, reproducible results in mechanistic studies and therapeutic trials. Consistent with the literature [59, 60], our study indicated that 1 µM DOX induced apoptosis in HL-1 cardiomyocytes, as evidenced by significant increases in the levels of various apoptosis markers (Figs. 1C, D). Moreover, annexin V staining revealed that 24-h DOX treatment substantially increased the level of apoptosis in cardiomyocytes, with apoptosis persisting even after 24 h following drug withdrawal. Quantitative analysis further confirmed that DOX significantly promoted apoptosis, which persisted even after DOX withdrawal (Fig. 1C). Specifically, the percentage of early apoptotic cells (annexin V^+^/PI^−^) was significantly higher in the DOX-treated cells than in control cells, both at 24 h after DOX treatment and 24 h after drug withdrawal. Notably, although pretreatment with DRZ reduced the level of apoptosis, it could not fully match the level in control cells; this finding indicates a partial protective effect of DRZ on cardiomyocytes. Western blotting revealed increased levels of cleaved caspase-3 after DOX treatment, which did not revert to baseline levels after drug withdrawal. These results indicate that DOX resulted in sustained apoptosis (Figs. 1D and S1). The ratio of cleaved caspase-3 to procaspase-3 was significantly higher in DOX-treated cells than in control cells. The DOX-induced increase in this ratio persisted even after drug withdrawal, suggesting that DOX led to persistent apoptosis in cardiomyocytes.

In summary, DOX caused significant and irreversible damage to cardiomyocytes, as evidenced by persistent loss of metabolic activity, induction of apoptosis, and activation of caspase-3, even after drug withdrawal. In subsequent experiments, we evaluated the efficacy of EVS_ASC_ in mitigating the cardiotoxicity of 1 µM DOX; this concentration induces a significant yet controllable level of apoptosis in cardiomyocytes. By administering DOX at this concentration, we sought to facilitate the development of effective cardioprotective strategies.

### ASCs reduces DOX-induced apoptosis and caspase-3 activation

**Fig. 2:**
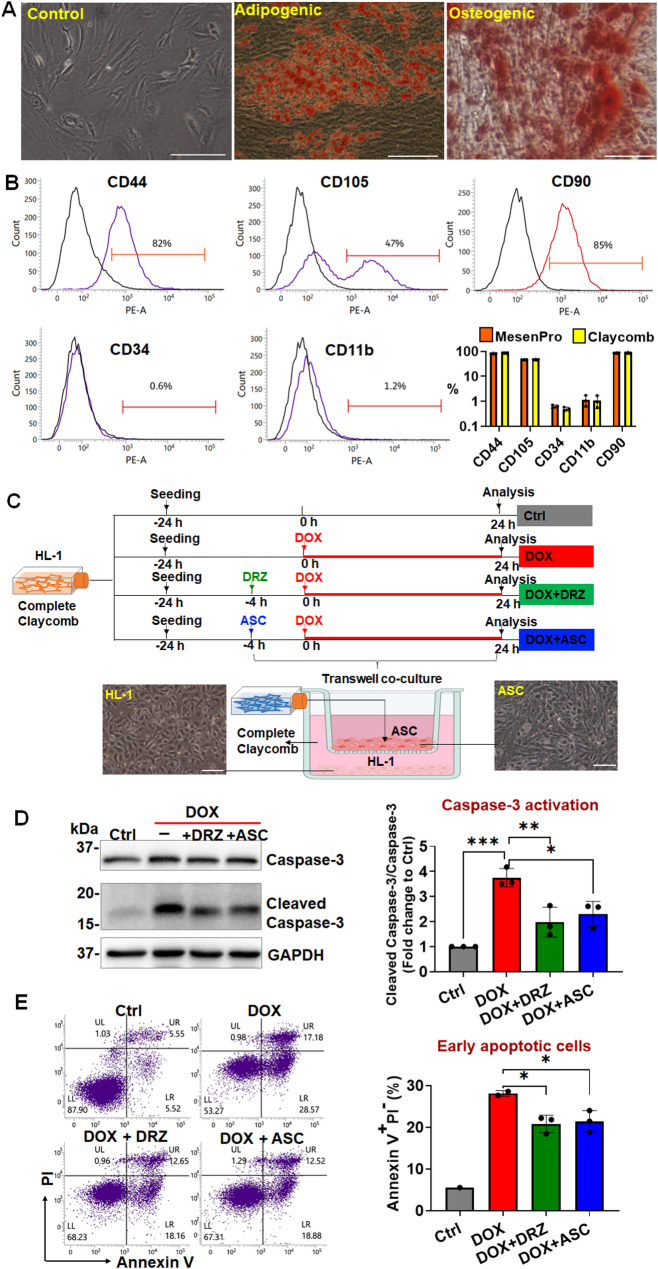
ASCs decreased doxorubicin-induced apoptosis and caspase-3 activation through paracrine effects in a Transwell coculture system **(A)** Differentiation of ASCs into adipocytes and osteocytes was confirmed using Oil Red O staining and Alizarin Red staining. Scale bar, 100 μm. **(B)** Comparing surface markers of ASCs in MesenPro medium and Complete Claycomb medium. **(C)** Schematic diagram of the Transwell coculture experimental timeline. HL-1 cardiomyocytes were seeded into the lower wells of a 6-well plate, and ASCs were seeded into the upper Transwell inserts. Both cell types were maintained in a Complete Claycomb medium (containing 10% FBS) throughout the experiment. After a 4-hour coculture adaptation period, DOX was added to induce cardiotoxicity. ASCs were retained in the upper chamber during DOX exposure to maintain continuous paracrine signaling and simulate coexposure conditions. Scale bar, 100 μm. **(D)** Caspase-3 activation was assessed in cardiomyocytes treated with 1 µM DOX alone or in coculture with ASCs using Western blotting. Cropped gels and blots are displayed, with full-length blots provided in Supplementary Fig. 2. **(E)** Quantitative analysis of annexin V-positive cells and cleaved caspase-3 bands normalized to GAPDH confirmed the statistically significant reduction in apoptosis and caspase-3 activation in the presence of ASCs compared to DOX treatment alone. *p < 0.05; **p < 0.01; ***p < 0.005

We assessed the differentiation ability and expression of ASC surface markers to determine the phenotypic profile of ASCs. Notably, ASCs can differentiate into both adipocytes and osteocytes (Fig. 2A). The phenotypic characterization confirmed the presence of expected surface markers such as CD44 (82%), CD105 (47%), and CD 90 (85%) but not CD34 (0.6%) or CD11b (1.2%; Fig. 2B). This finding aligns with that reported by another study [52]. Subsequently, we evaluated the effects of ASCs on DOX-treated HL-1 cardiomyocytes. For this, the cardiomyocytes were cultured with 1 µM DOX alone or cocultured with ASCs in a Transwell system for 24 h (Fig. 2C). This system (pore size: 0.4 μm) allowed the passage of ASC-secreted soluble factors, thereby promoting paracrine signaling without direct cell–cell contact.

Culture media requirements differ between ASCs (i.e., MesenPro medium) and HL-1 cardiomyocytes (i.e., Complete Claycomb medium). Thus, to ensure medium compatibility, we compared the expression levels of surface markers between ASCs cultured in MesenPro medium and those cultured in Complete Claycomb medium. Transitioning from the MesenPro medium to the Complete Claycomb medium did not significantly alter the surface marker profile of ASCs (Fig. 2B). The expression levels of CD44, CD105, CD90, CD34, and CD11b were similar in the two media. This result indicates that the culture medium did not significantly affect the phenotypic characteristics of ASCs and confirmed the suitability of using the Complete Claycomb medium in subsequent experiments.

To evaluate the protective effects of ASCs against DOX-induced apoptosis in HL-1 cardiomyocytes, we evaluated caspase-3 activation in these cells through Western blotting. A significant reduction was noted in the level of DOX-induced caspase-3 activation in HL-1 cardiomyocytes cocultured with ASCs (Figs. 2D and S2), which indicated that ASCs exert protective effects similar to those of DRZ. Densitometric analysis revealed a significant reduction in the cleaved caspase-3–procaspase-3 ratio in HL-1 cardiomyocytes cocultured with ASCs, indicating reduced apoptosis (Figs. 2D and S2). Next, the number of apoptotic cells was quantified through annexin V staining. The number of annexin V^+^ cells was significantly lower in DOX-treated HL-1 cells cocultured with ASCs than in those cultured alone. Quantitative analysis of early apoptotic cells (annexin V^+^/PI^−^) confirmed a significant reduction of apoptosis in the cardiomyocytes cocultured with ASCs (Fig. 2E). These findings indicate that ASCs protect against DOX-induced apoptosis through paracrine signaling, suggesting that ASC-derived secretome is a viable cardioprotective strategy.

### CM protects HL-1 cardiomyocytes from DOX-induced apoptosis

**Fig. 3:**
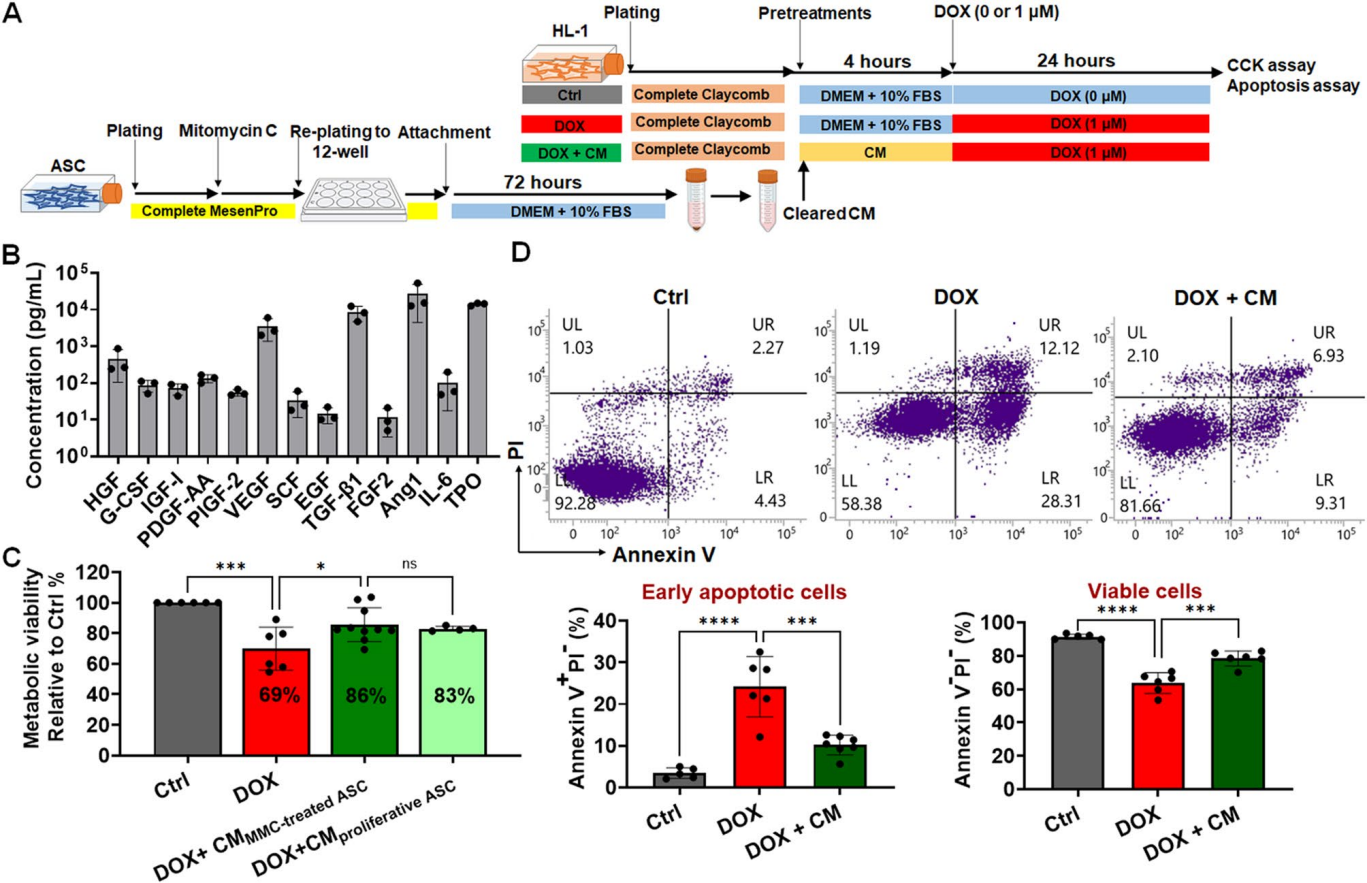
CM attenuated DOX-induced apoptosis in cardiomyocytes **(A)** The schematic illustrates the timeline of cell seeding, coculture, and treatment phases. CM was prepared by culturing ASCs in DMEM-HG supplemented with 10% standard FBS for 72 h, followed by filtration. All DOX-only and control groups were cultured in DMEM-HG medium containing 10% FBS to confirm that CM-specific effects were not due to FBS. **(B)** Adipose-derived stem cells (ASCs) secrete a range of pro-angiogenic and cardioprotective factors, including hepatocyte growth factor (HGF), granulocyte colony–stimulating factor (G-CSF), insulin-like growth factor 1 (IGF-I), platelet-derived growth factor (PDGF), placental growth factor (PIGF), vascular endothelial growth factor (VEGF), stem cell factor (SCF), epidermal growth factor (EGF), transforming growth factor (TGF-β1), fibroblast growth factor-2 (FGF-2), angiopoietin-1 (Ang1), interleukin (IL)-6, and thrombopoietin (TPO). **(C)** HL-1 cardiomyocytes were pretreated with CM derived from either mitomycin C-treated ASCs (CM _MMC−treated ASC_) or untreated ASCs (CM _proliferative ASC_) for 4 h prior to doxorubicin (DOX, 1 µM) exposure, and CM was replenished during the 24-hour DOX treatment. Cell viability was assessed using the CCK-8 assay. **(D)** The impacts of ASC-derived CM on DIC, as determined by annexin V and propidium iodide (PI) staining, highlight its role in reducing cardiomyocyte apoptosis. *p < 0.05; ***p < 0.005; ****p < 0.0001

On the basis of the results of the Transwell assay, we evaluated the therapeutic potential of the CM collected from a culture of ASCs (Fig. 3A). The CM was collected at 72 and 120 h during cell culture (Fig. S3A). It contained a diverse array of ASC-secreted bioactive compounds (Fig. 3B; Table S1), such as granulocyte colony–stimulating factor [61] and thrombopoietin [62], which are both known for their cardioprotective properties.

We then compared the levels of various growth factors in the CM between the medium collected at 72 h and that collected at 120 h. In general, the levels of growth factors were higher in the CM collected at 120 h than in that collected at 72 h (Fig. S3B). The CM collected at 72 h and that collected at 120 h significantly restored the metabolic activity of DOX-treated HL-1 cardiomyocytes to 82% and 86%, respectively, of that noted in cardiomyocytes treated with DOX alone (Fig. S3C). Given the similar efficacy between the media collected at the two time points and the short duration (72 h) of CM preparation, we selected the 72-h CM for subsequent experiments. The 72-h protocol ensured efficient CM production while maintaining its therapeutic efficacy.

The 72-h CM restored viability and significantly reduced apoptosis in DOX-treated cardiomyocytes; this finding highlights the CM’s potential for mitigating DOX-induced apoptosis (Figs. 3C, D). The CCK-8 assay confirmed that the CM restored the viability of HL-1 cardiomyocytes (Fig. 3C). Annexin V and PI staining revealed a substantial reduction in apoptosis (Fig. 3D). Specifically, the percentage of early apoptotic cells (annexin V^+^/PI^−^) was considerably lower in the DOX + CM group than in the DOX-only group (Fig. 3D). This result confirmed the protective effects of the CM against DOX-induced apoptosis.

### Functional characteristics of CM-derived EVs

**Fig. 4:**
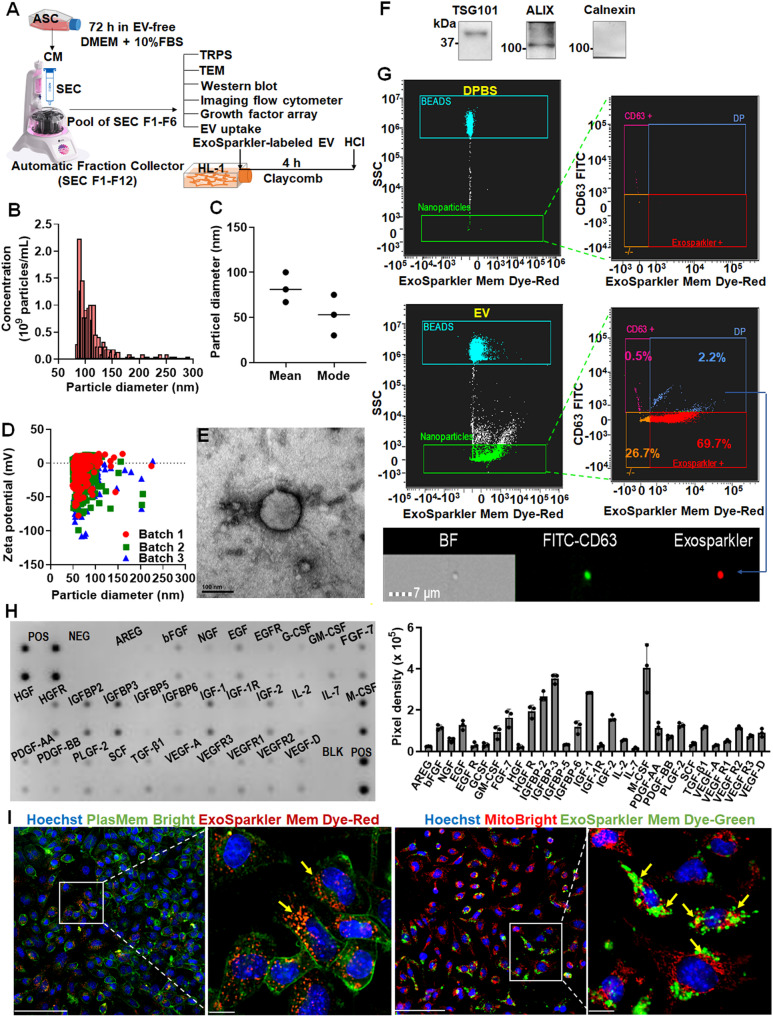
EV fulfilled with Minimal Information for Studies of Extracellular Vesicles 2023 guidelines interacts with cardiomyocytes **(A)** Schematic representation of the isolation of extracellular vesicles (EVs) using the combination of qEV size exclusion columns (SEC) and an automatic fraction collector, followed by characterization and tracking of EVs in HL-1 cardiomyocytes. The first six fractions (F1-F6) after the buffer were collected, with each fraction being 0.7 mL. **(B)** Simultaneous measurement of size and concentration using tunable resistive pulse sensing (TRPS). **(C)** Statistical analysis of particle diameters. **(D)** Concurrent measurement of both size and zeta potential via TRPS. **(E)** Transmission electron microscopy (TEM) images. **(F)** Detection of internal markers; cropped gels and blots are shown, with full-length blots available in Supplementary Fig. 4. **(G)** Profiling of the exosomal marker CD63 within EVs and Dulbecco’s Phosphate-Buffered Saline (DPBS). **(H)** Profiles of growth factors present in EVs. **(I)** Interaction and uptake of EVs by HL-1 cardiomyocytes, emphasizing their proximity to mitochondria. Two co-staining protocols were utilized: the first employed Hoechst for nuclei, PlasMem for cell membranes, and ExoSparkler for exosomal membranes; the second used Hoechst for nuclei, MitoBright for mitochondria, and ExoSparkler for exosomal membranes. Colocalization of EVs with mitochondrial markers suggests their involvement in modulating mitochondrial function and oxidative stress in cardiomyocytes, providing insights into potential therapeutic mechanisms against DIC. Scale bar, 100 μm. Magnified view of the white-boxed region. Scale bar, 10 μm. HCI = High-Content Imaging System. SEC = size exclusion chromatography

We isolated EVs from the CM and characterized them to assess their specific contributions to the observed benefits of the CM (Fig. 4A). To isolate EVs from the CM, ASCs were cultured in EV-free media for 72 h. The supernatants were concentrated, and EVs were separated through size exclusion chromatography. An automatic fraction collector was used to separate and collect fractions from the column, distinguishing between the buffer volume and the EV eluent. Most EVs elute in 3.5 mL, corresponding to the first four fractions following the buffer. However, to maximize recovery, we collected a volume of 4.2 mL, corresponding to the first six fractions after the buffer, with each fraction measuring 0.7 mL (Fig. 4A). The isolated EVs were characterized following the Minimal Information for Studies of Extracellular Vesicles 2023 guidelines [63]. Their size, concentration, and zeta potential were evaluated using tunable resistive pulse sensing technology. The results revealed that the concentration was within the expected range (Fig. 4B) and that the predominant size was approximately 100 nm (Fig. 4C), indicating a relatively homogeneous population of small EVs. The average zeta potential of different batches of EVs in PBS ranged from − 20 to − 7 mV (Fig. 4D). Transmission electron microscopy confirmed the characteristic spherical and cup-shaped morphology of the EVs (Fig. 4E), which was consistent with that of small EVs.

The expression profiles of EV-specific markers were analyzed. Western blotting revealed the presence of the endosomal markers TSG101 and ALIX in the EVs, confirming that the EVs had an endosomal origin. The absence of calnexin, an endoplasmic reticulum marker, in the EVs suggested minimal contamination with other cellular components, indicating the purity of the isolated EVs (Figs. 4F and S4). Flow cytometry revealed that a subpopulation of EVs (2.2%) was positive for both CD63 and ExoSparkler dye (Fig. 4G). No EVs were detected in Dulbecco’s PBS.

A growth factor array was performed for the functional characterization of the isolated EVs. The results revealed the presence of cardioprotective factors in these EVs. The array detected significantly high levels of key growth factors, such as macrophage colony–stimulating factor, epidermal growth factor, and insulin-like growth factor-1 (Fig. 4H). These factors have been demonstrated to mitigate DIC [64–66]. Therefore, this finding indicates that the ASC-derived EVs contained abundant bioactive compounds with therapeutic potential against DIC.

### Intracellular Tracking Reveals Mitochondrial Localization of EVs in HL-1 Cardiomyocytes

We hypothesized that EVs would be localized near the mitochondria in HL-1 cardiomyocytes to exert their antiapoptotic effects. To test this hypothesis, 2 × 10^4^ HL-1 cells were incubated with 30 µg of EVs (approximately 10^9^ particles) in a 96-well plate. Two costaining protocols were used to visualize the localization of the EVs in HL-1 cardiomyocytes. In the first protocol, Hoechst (blue), PlasMem Bright (green), and ExoSparkler Mem Dye-Red (red) were used to stain the nuclei, cell membranes, and exosomal membranes, respectively. The results indicate that the ASC-derived EVs were internalized by HL-1 cells, as evidenced by the red fluorescence within the cells (Fig. 4I, left panel). Thus, the first protocol effectively visualized the localization of the EVs relative to the cellular membrane, confirming the internalization of the EVs.

In the second protocol, Hoechst (blue), MitoBright (red), and ExoSparkler Mem Dye-Green (green) were used to stain the nuclei, mitochondria, and exosomal membranes, respectively. The colocalization of green and red fluorescence indicated that the ASC-derived EVs were localized near the mitochondria in HL-1 cardiomyocytes (Fig. 4I, right panel). The results provided detailed insights into the intracellular distribution of EVs, revealing their close association with the mitochondria. The colocalization of EVs with the aforementioned dyes that stain the mitochondria suggests that these vesicles reduce apoptosis and promote cardiomyocyte survival through their influence on the mitochondria.

### CM-derived EVs protect against DIC, potentially by preventing mitochondrial damage and enhancing cell survival

**Fig. 5:**
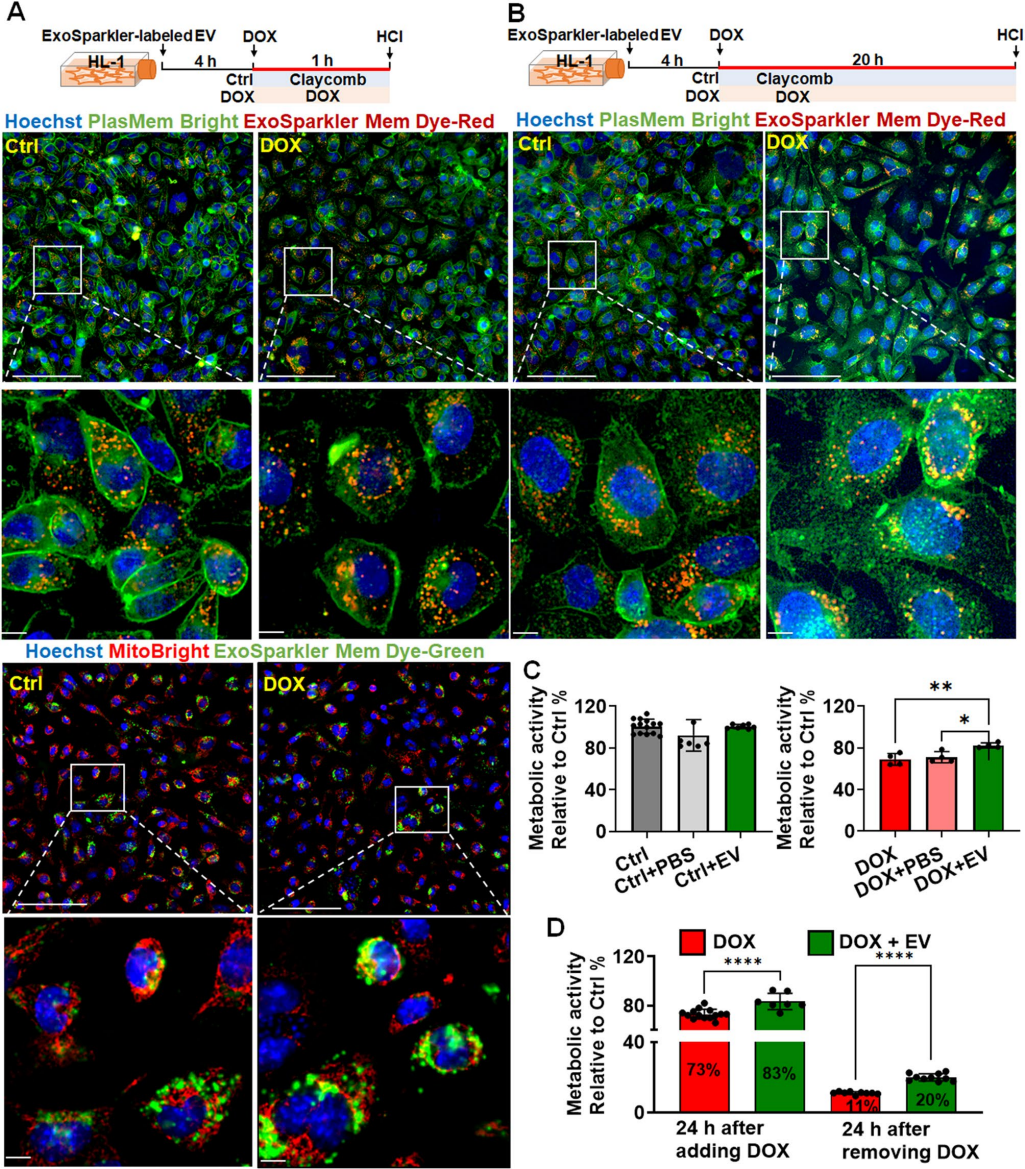
EV restored the viability of HL-1 cardiomyocytes post-DOX exposure **(A)** Interaction and uptake of extracellular vesicles (EVs) by HL-1 cardiomyocytes 1 h after DOX exposure, compared to control cells, demonstrating initial EV internalization. **(B)** Interaction and uptake of EVs by HL-1 cardiomyocytes 20 h after DOX exposure, compared to control cells, indicating sustained EV presence and cellular uptake over time. **(C)** Impacts of EVs and their vehicle, PBS, on control cells and DOX-treated cells, providing a baseline for EV interaction in the absence of DOX-induced stress. **(D)** Effects of EV treatment on cardiomyocyte survival at the end of the 24-hour DOX exposure period and 24 h after DOX removal, highlighting the sustained cardioprotective effects of EVs in mitigating DOX-induced cytotoxicity. HCI = High-Content Imaging (HCI) System. * *p* < 0.05; ** *p* < 0.01; **** *p* < 0.0001. Scale bar, 100 μm. Magnified view of the white-boxed region. Scale bar, 10 μm

DOX-treated cardiomyocytes were incubated with CM-derived EVs. Despite the induction of oxidative stress by DOX, the EVs were successfully internalized by HL-1 cardiomyocytes, as evidenced by their localization near the mitochondria. This finding indicates the protective effects of EVs (Fig. 5A). The early internalization of the EVs—1 h after DOX exposure—confirmed the rapid uptake of these vesicles by cardiomyocytes. Thus, these findings indicate that EVs can rapidly enter the mitochondria under chemotherapeutic stress.

The EVs were internalized 20 h after DOX exposure, indicating the persistent interaction and localization of EVs within HL-1 cardiomyocytes (Fig. 5B). The prolonged interaction highlighted the persistent presence of EVs and their continued association with the mitochondria. This finding suggests that EVs have a sustained capacity to mitigate DOX-induced mitochondrial damage and enhance cell survival.

To establish a baseline for the interaction of EVs with HL-1 cardiomyocytes in the absence of DOX-induced stress, we analyzed the effects of EVs and their vehicle on untreated cardiomyocytes (Fig. 5C, left panel). The results served as a reference for the unstressed condition, demonstrating the natural behavior and localization patterns of EVs in untreated cardiomyocytes. The findings confirmed that EVs did not adversely affect the metabolic activity of normal cardiomyocytes (Fig. 5C, left panel). With this baseline established, we assessed the effects of EVs on the survival of cardiomyocytes at 24 h after DOX exposure and 24 h after DOX withdrawal. After 24-h treatment, the metabolic activity of the DOX-treated cells decreased to 73% of the baseline activity. Cotreatment with DOX and EVs significantly increased the cells’ metabolic activity to 83%, whereas treatment with PBS alone exerted no significant effects (Fig. 5C, right panel). After 24 h following DOX withdrawal, the metabolic activity of the DOX-treated cells markedly decreased to 11% of the baseline level. Cotreatment with DOX and EVs significantly increased metabolic activity to 20% (Fig. 5D). These findings highlight the protective effects of CM-derived EVs against DOX-induced DIC.

### Clusterin expression is upregulated in DOX- and CM-treated HL-1 cardiomyocytes

We further investigated the mechanisms underlying the cardioprotective effects of ASCs by analyzing DEGs in HL-1 cardiomyocytes incubated with or without the CM. This approach was adopted to comprehensively understand the therapeutic effects of ASCs.

**Fig. 6:**
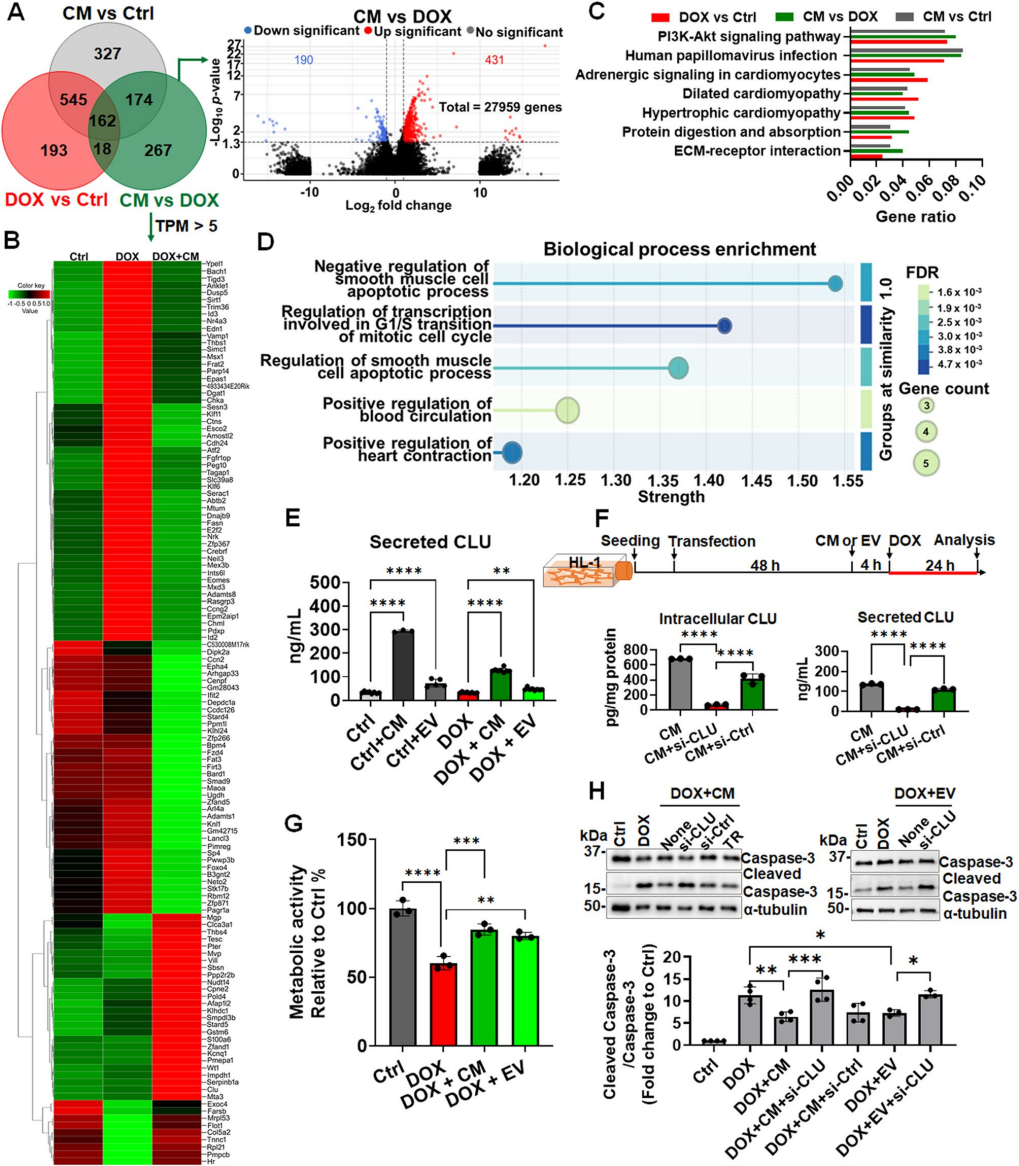
Clusterin played a critical role in CM and EVs against DOX-induced apoptosis **(A)** Venn diagram of differentially expressed genes (DEGs) across three experimental groups. A total of 621 genes uniquely modulated by CM in response to DIC are highlighted within the green circle. The accompanying volcano plot illustrates the statistical significance and magnitude of changes in the expression of these 621 genes, facilitating the rapid identification of significant DEGs. **(B)** Heat maps of protein-coding DEGs with TPM values above 5 in the CM-treated group are clustered hierarchically to reveal expression patterns across samples. **(C)** KEGG pathway analysis highlights representative pathways featuring the top 7 gene ratios. Gene ratio (x-axis) is the percentage of the number of genes present in this GO term over the total number of genes in this category. **(D)** The top 5 terms from the functional analysis of biological processes based on false discovery rate (FDR) in the CM-treated group are presented. **(E)** The quantification of clusterin (CLU) levels in HL-1 cardiomyocyte lysates and supernatants shows an upregulation following CM or EV treatment. **(F)** A schematic representation explains the siRNA-mediated knockdown of clusterin, outlining the experimental workflow, including quantification of clusterin in HL-1 cardiomyocyte lysates (intracellular CLU) and supernatant (secreted CLU) post-siRNA treatment, confirming effective knockdown. **(G)** Metabolic activity assessments reveal comparable protective effects of CM and EVs, as demonstrated by CCK-8 assays. **(H)** A representative Western blot shows caspase-3 activation, illustrating the protective effect of CM and EV treatment. Cropped gels and blots are shown, with the full-length blots available in Supplementary Fig. 6. Densitometric analysis of Western blot data provides statistical validation. * *p* < 0.05; **p < 0.01; ***p < 0.005; ****p < 0.0001. si-CLU = siRNA targeting clusterin; si-Ctrl = negative control siRNA; TR = Lipofectamine RNAiMAX Transfection Reagent. None = no siRNA or TR treatment

To systematically elucidate the mechanisms underlying the cardioprotective effects of the CM, we first performed RNA-seq to identify DEGs in DOX-treated HL-1 cardiomyocytes incubated with or without the CM. The Venn diagram in Fig. 6A depicts the DEGs across three experimental groups: DOX/control (red circle, totaling 918 DEGs), CM/DOX (green circle, totaling 621 DEGs), and CM/control (gray circle, totaling 1208 DEGs). A total of 162 DEGs were shared among the 3 groups. The volcano plot in Fig. 6A (right panel) presents the significance and magnitude of the CM-induced changes in the expression levels of the 621 DEGs in the CM/DOX group.

To identify the specific gene expression patterns, we generated heat maps through hierarchical clustering. The heat maps depicted the expression profiles of protein-coding genes with a TPM value of > 5 across the following three experimental conditions: control, DOX-only, and DOX + CM. Distinct clusters of upregulated and downregulated genes were observed in the heat maps (Fig. 6B). Among the 126 protein-coding DEGs identified on the basis of the CM-to-DOX ratio of TPM values, 35 genes were upregulated, whereas 91 were downregulated (Fig. 6B; Table S2). The baseline gene expression levels in the control group were used as a reference for comparing the effects of various treatments on gene expression.

The DOX group exhibited marked upregulation of several genes, as indicated by a shift toward red in the heat map. This finding reflected significant changes in gene expression levels due to DOX-induced stress. For example, the expression of genes such as *Edn1* and *Ccng2* was upregulated in the DOX group. However, in the DOX + CM group, the expression of these genes was downregulated to levels similar to those observed in the control group. Therefore, CM treatment likely reversed some of the DOX-induced changes in gene expression. Notably, the expression levels of *S100a6* and *Clu* were higher in the DOX + CM group than in the control and DOX groups. CM treatment upregulated the expression of these genes, likely to improve cardiomyocyte survival and function. Notably, *Clu* exhibited the highest TPM value among the identified DEGs (control = 362, DOX = 431, and DOX + CM = 1286; Table S2), with a fold change of approximately 2.9. The substantial increase in *Clu* expression after CM treatment indicates that the corresponding protein mediates the cardioprotective effects of the CM.

The Kyoto Encyclopedia of Genes and Genomes analysis indicated that the DEGs between the DOX + CM and DOX groups were enriched in several pathways, with the PI3K/AKT pathway exhibiting a notably high gene ratio (Figs. 6C and S5A). For Gene Ontology functional analysis, AKT was included alongside the 126 DEG-encoded proteins in the Search Tool for the Retrieval of Interacting Genes/Proteins database. The analysis revealed several biological processes that were influenced by CM treatment—for example, smooth muscle cell apoptosis, blood circulation, and heart contraction (Figs. 6D and S5B).

### Clusterin knockdown reduces the protective effects of CM against DOX-induced apoptosis

To validate our RNA-seq data, we performed enzyme-linked immunosorbent assay, quantifying the expression of clusterin in CM- or EV-treated HL-1 cardiomyocytes (Figs. 6E, F). Clusterin is a multifunctional protein known for the roles it plays in cell protection and apoptosis inhibition. Both the CM and the EVs upregulated the expression of clusterin in DOX-treated cardiomyocytes (Fig. 6E). Notably, even in the absence of DOX, the expression of clusterin remained upregulated in CM- or EV-treated cardiomyocytes (Fig. 6E), suggesting that EVS_ASC_ serves as a preconditioning agent, priming cells to better withstand future stress. Although the CM and EVs exerted similar restoring effects on the metabolic activity of HL-1 cells (Fig. 6G), differences were noted in the level of clusterin expression: the CM and EVs induced the cells to secrete clusterin at concentrations of approximately 127.2 ± 8.4 and 46.9 ± 5.5 ng/mL, respectively.

To confirm the functional significance of clusterin in CM-mediated cardioprotection, we knocked down clusterin expression in HL-1 cardiomyocytes. Notably, siRNA-mediated knockdown effectively downregulated clusterin expression in CM-treated HL-1 cells, as evidenced by significant reductions in the levels of clusterin protein in both the cell lysate and supernatant (Fig. 6F).

After clusterin knockdown, the HL-1 cells were treated with the CM or EVs and then with DOX. Clusterin knockdown significantly reduced the protective effects of the CM or EVs, as evidenced by a marked increase in caspase-3 activation compared with that in cells transfected with nontargeting siRNA (Figs. 6H and S6). Western blotting revealed elevated levels of cleaved caspase-3 in cells with clusterin knockdown, indicating increased apoptosis. Thus, clusterin knockdown led to the loss of the antiapoptotic effects of the CM or EVs. Therefore, clusterin plays a key role in mediating the cardioprotective effect of EVS_ASC_. This finding was confirmed by the densitometric analysis of the results of Western blotting, which indicated a significant increase in the ratio of cleaved caspase-3 to procaspase-3 in cells with clusterin knockdown (Fig. 6H, lower panel). The significant increase in this ratio highlights the vital role of clusterin in mitigating DOX-induced apoptosis, underscoring its importance in the therapeutic potential of EVS_ASC_.

### CM mitigates DOX-induced apoptosis by activating PI3K/AKT signaling and reducing mitochondrial superoxide production

**Fig. 7:**
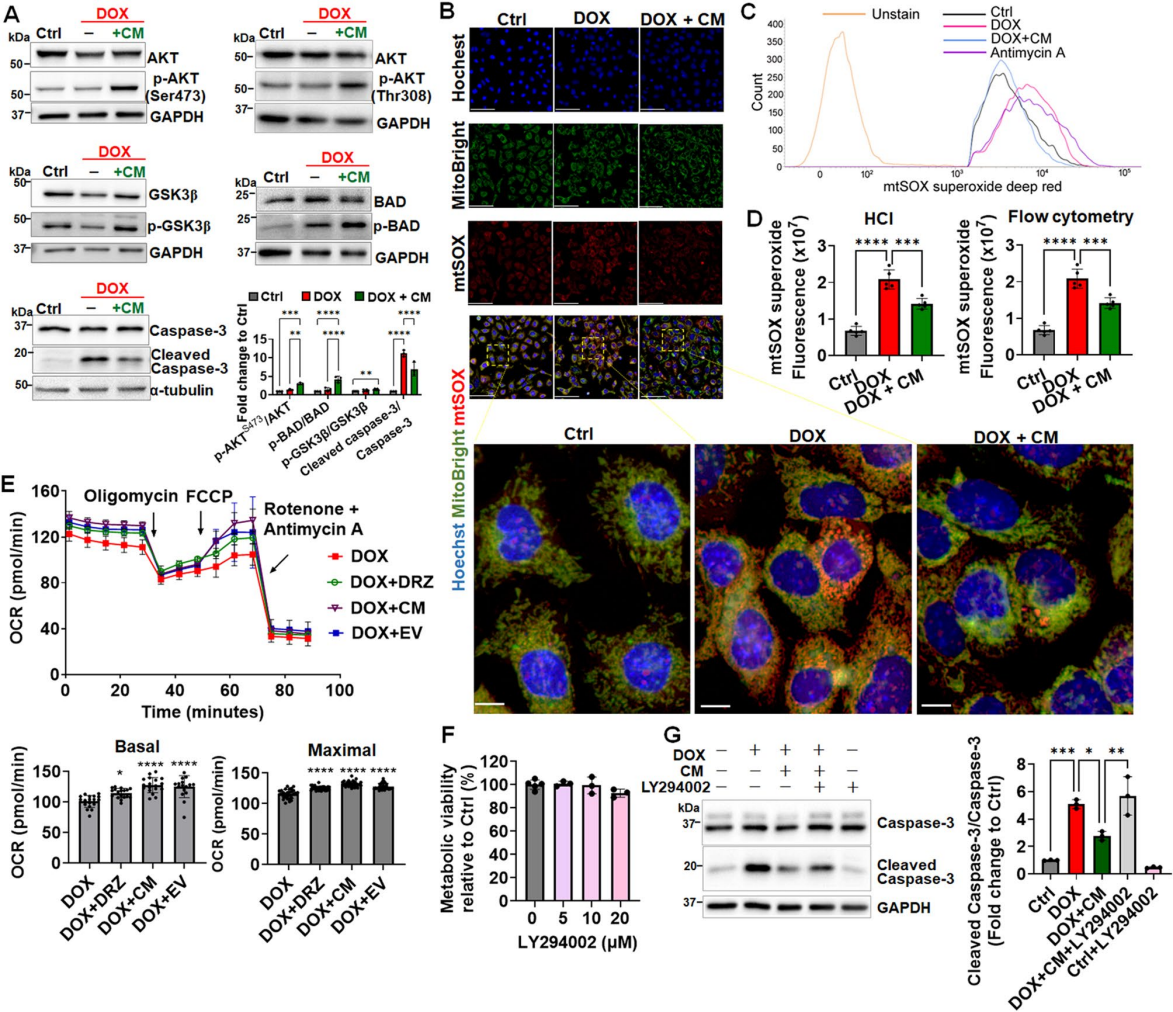
CM activated AKT and reduced mitochondrial superoxide production **(A)** Representative Western blot images illustrating the levels of caspase-3 activation alongside the phosphorylation of AKT, BAD, and GSK3β in response to CM treatment, highlighting the regulatory effects of CM on AKT-BAD and AKT-GSK3β signaling. Cropped gels and blots are shown, with full-length blots available in Supplementary Fig. 7. Densitometric analysis of the Western blot results provides quantitative validation of the observed protein expression changes. **(B)** Representative high-content images (HCI) show co-staining for Hoechst (nuclei), MitoBright (mitochondria), and mtSOX (mitochondrial superoxide), along with merged panels that illustrate cellular localization and oxidative stress response. Enlarged images from the boxed regions highlight the colocalization of mitochondria and oxidative stress markers, providing deeper insight into the intracellular effects of CM treatment. Scale bar, 100 μm. Magnified view of the white-boxed region. Scale bar, 10 μm. **(C)** A representative histogram of flow cytometry analysis displays the fluorescence of mtSOX superoxide. **(D)** Quantitative analysis of mtSOX fluorescence intensity is conducted using both HCI and flow cytometry to assess oxidative stress levels in cardiomyocytes across different treatment conditions. **(E)** Oxygen consumption rate (OCR) measurement by using the Seahorse XFe96 extracellular flux analyzer at baseline and after the addition of oligomycin, carbonyl cyanide-4-(trifluoromethoxy) phenylhydrazone (FCCP), and rotenone + antimycin A. Mitochondrial function, focusing on basal and maximal respiration in HL-1 cardiomyocytes pretreated with DRZ, CM, or EV, was compared to groups treated only with DOX. **(F)** Control experiments verified that treatment with LY294002 alone, ranging from 5 to 20 µM, did not induce significant cytotoxicity. **(G)** HL-1 cells were pretreated with LY294002 (20 µM) for 2 h before the addition of CM and/or DOX. After pretreatment, cells were exposed to DOX (1 µM) and CM for 24 h. Caspase-3 cleavage was subsequently assessed by Western blotting to evaluate apoptosis. The experimental groups included: (1) Control (Ctrl), (2) DOX-only, (3) DOX + CM, (4) DOX + CM + LY294002, and Ctrl + LY294002. *p < 0.05; **p < 0.01; ***p < 0.005; ****p < 0.0001

We investigated whether the clusterin-mediated cardioprotective effects of the CM involve the PI3K/AKT pathway (Fig. 6C), which is known to enhance cell survival and growth [67, 68]. The AKT pathway plays a crucial role in protecting cells from apoptosis. BAD, a proapoptotic member of the Bcl-2 family, and GSK3β are inactivated through AKT-mediated phosphorylation [21, 22, 67]. Phosphorylated BAD is sequestered by 14-3-3 proteins, resulting in the suppression of apoptosis [22]. When AKT inactivates GSK3β, it dephosphorylates and inactivates BAX, further inhibiting apoptosis [69]. To investigate whether the CM’s cardioprotective effect involves activation of the AKT/BAD or AKT/GSK3β pathway, we performed Western blotting targeting key phosphorylated proteins. Specifically, HL-1 cardiomyocytes were treated with the CM under DOX-induced stress conditions, and the levels of phosphorylated AKT (at both Ser473 and Thr308), BAD, and GSK3β were evaluated. Our results reveal that CM treatment markedly increased the levels of phosphorylated AKT (at both Ser473 and Thr308), BAD, and GSK3β compared with the corresponding levels in DOX-treated cells (Figs. 7A and S7). The phosphorylation events were associated with significant reductions in cleaved caspase-3 levels, indicating reduced apoptosis and enhanced cardiomyocyte survival. Densitometric analysis of the immunoblots further confirmed these observations. Phosphorylation ratios (phosphorylated protein/total protein) of AKT (Ser473), BAD, and GSK3β were significantly elevated after CM treatment. Simultaneously, caspase-3 activation was suppressed after CM treatment (Figs. 7A and S7). Collectively, these findings suggest that the CM protects HL-1 cardiomyocytes, at least partially, by activating the PI3K/AKT pathway and its downstream antiapoptotic effectors.

We measured mitochondrial superoxide levels in DOX-treated HL-1 cardiomyocytes to investigate whether CM mitigates oxidative stress, a key driver of DOX-induced cardiomyopathy [15]. Costaining with Hoechst (nuclei), MitoBright (mitochondria), and mtSOX (mitochondrial superoxide) revealed the presence of superoxide ions within the mitochondria (Fig. 7B) of DOX-treated cells. Flow cytometry confirmed that the mean fluorescence intensity shifted to the right upon DOX exposure but returned to the baseline level after CM treatment (Fig. 7C). Statistical analysis of the fluorescence intensity of mtSOX indicated that CM treatment significantly reduced mitochondrial superoxide levels (Fig. 7D). Flow cytometry and confocal imaging provided complementary insights into the ROS dynamics. Flow cytometry facilitated a robust, quantitative assessment of mitochondrial superoxide ions, whereas confocal microscopy captured the spatial distribution of these ions within the mitochondria—information that cannot be discerned through flow cytometry alone. Adopting this integrated approach helped us comprehensive understand ROS localization and modulation under the experimental conditions of this study.

The protective effects of EVS_ASC_ extended beyond oxidative stress reduction. RNA-seq analysis (Fig. 6B; Table S2) unveiled several DEGs associated with mitochondrial function and bioenergetics whose expression levels were modulated in response to CM treatment. These DEGs included *Ppp2r2b* (2.28-fold increase), *Pmpcb* (2.36-fold increase), and *Mrpl53* (2.19-fold increase), which are involved in mitochondrial biogenesis and translation. The CM downregulated genes such as *Ctns* (0.47-fold reduction), *Atf2* (0.43-fold reduction), and *Slc39a8* (0.48-fold reduction), which reduce mitochondrial efficiency or contribute to oxidative stress. Consistent with these transcriptomic findings, OCR measurements indicated that CM or EV treatment improved mitochondrial function, as evidenced by enhanced basal respiration and maximal respiration (Fig. 7E). Therefore, EVS_ASC_ not only reduces oxidative stress but also supports mitochondrial bioenergetics and functionality at both the transcriptional and the metabolic level.

To elucidate the mechanism through which CM confers protection against DOX-induced apoptosis, we inhibited the PI3K/AKT pathway. Treatment with LY294002 (20 µM), an inhibitor of the PI3K/AKT pathway, led to no significant cytotoxicity in HL-1 cardiomyocytes under control conditions (Fig. 7F). Cotreatment with DOX, CM, and LY294002 significantly attenuated the CM-mediated reduction in cleaved caspase-3 level compared with that in the DOX + CM group (Figs. 7G and S7). These findings indicate that inhibition of the PI3K/AKT pathway weakened the protective effects of the CM, confirming that this pathway mediates the CM’s antiapoptotic effects.

**Fig. 8:**
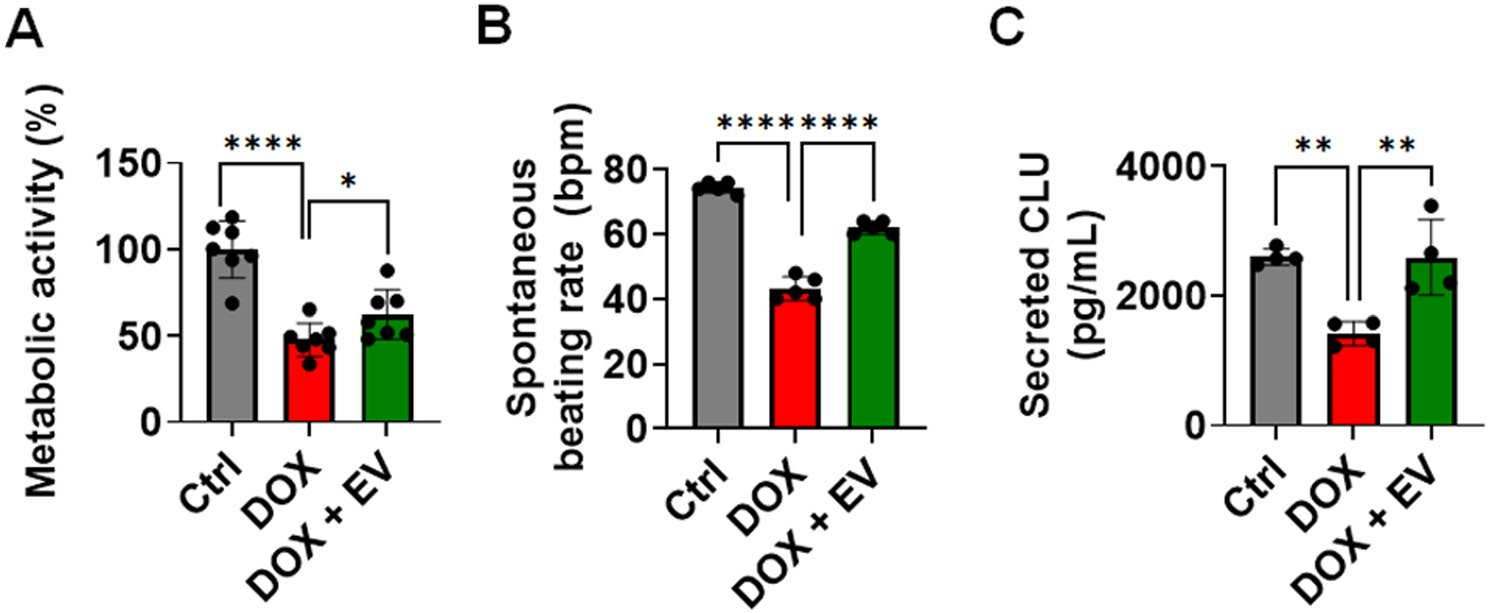
EVs restored metabolic activity and clusterin expression in induced pluripotent stem cell-derived human cardiomyocytes exposed to DOX treatment **(A)** Metabolic activity of cardiomyocytes was evaluated using the CCK-8 assay. **(B)** Spontaneous beating rates of human cardiomyocytes were recorded using an inverted phase-contrast microscope equipped with a high-resolution digital camera. **(C)** Clusterin expression levels were quantified via ELISA. *p < 0.05; **p < 0.01; ****p < 0.0001

Finally, we used hiPSC-derived cardiomyocytes as a functional model [70] to more accurately evaluate the cardioprotective effect of EVs. The CCK-8 assay revealed that EVs significantly improved the metabolic activity of DOX-treated cells, increasing viability from 48 to 62% (Fig. 8A), and preserved their beating rates (Fig. 8B). DOX significantly reduced clusterin secretion in hiPSC-derived cardiomyocytes from 2601 to 1423 pg/mL (Fig. 8C). EV treatment counteracted this reduction, restoring clusterin secretion to 2593 pg/mL (Fig. 8C). These results provide additional evidence for the protective effects of EVS_ASC_ in a human cell model, supporting its therapeutic potential against DIC.

## Discussion

**Fig. 9:**
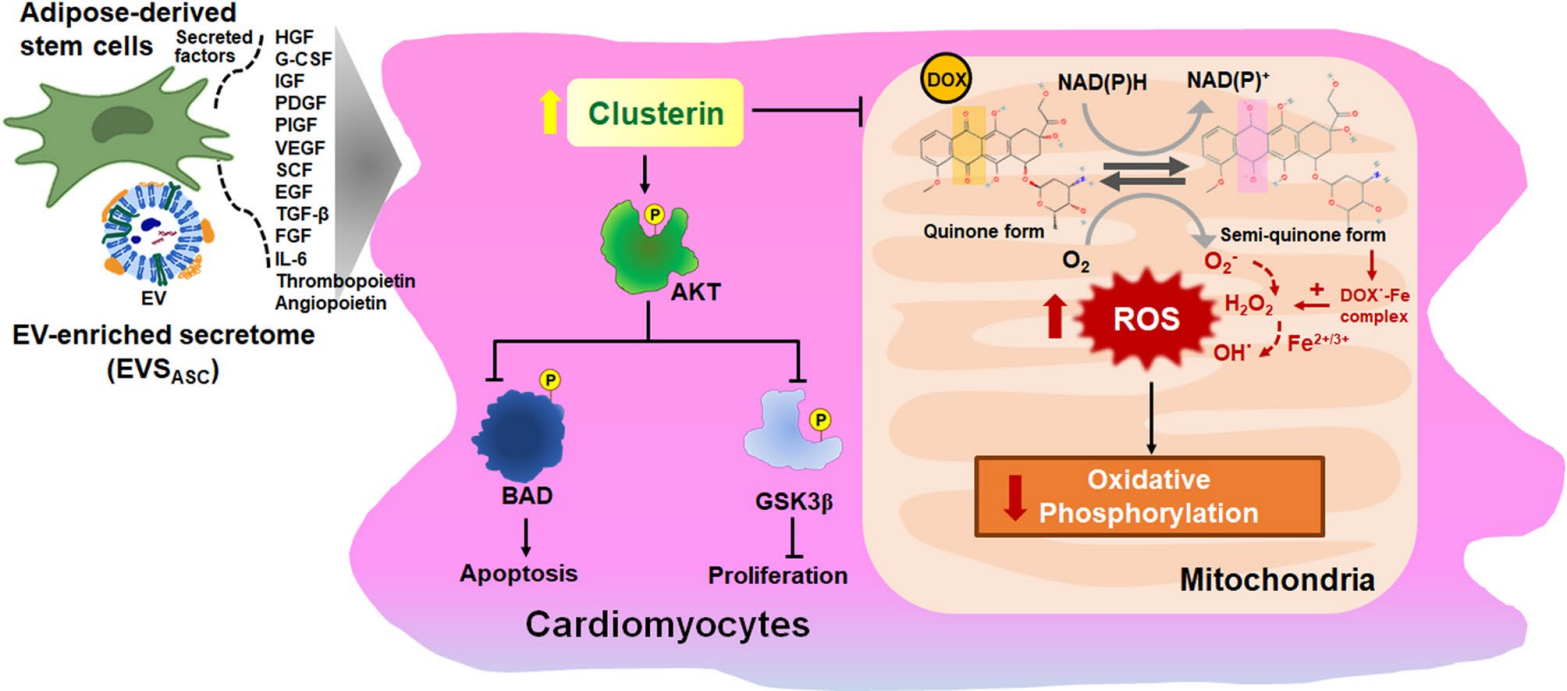
Schematic representation of the EV-enriched secretome from ASCs, which modulates mitochondrial ROS, reduces apoptosis, and increases clusterin in cardiomyocytes treated with DOX

In this study, the CM and EVs derived from ASCs were collectively referred to as EVS_ASC_. The CM contained a wide array of secreted factors, such as EVs, soluble proteins, growth factors, cytokines, and other bioactive compounds. EVs represented the purified vesicular component isolated from this complex mixture. Our findings underscore the therapeutic potential of EVS_ASC_ in attenuating DIC. Notably, EVS_ASC_ treatment restored cardiomyocyte viability and reduced the ratio of cleaved caspase-3 to procaspase-3 in DOX-treated HL-1 cells, thereby suppressing apoptosis. These protective effects were mediated by the PI3K/AKT pathway. Furthermore, clusterin emerged as a promising biomarker of EVS_ASC_-mediated cardioprotection (Fig. 9). This finding provides valuable insights into the molecular mechanisms underlying the therapeutic efficacy of EVS_ASC_.

Clusterin is a multifunctional glycoprotein [71] recognized for its protective effects in response to cardiac injury [67]. The roles of clusterin in myocardial infarction [72–75], myocarditis [76], and vascular injury [77] have been extensively studied. Mechanistically, clusterin protects cardiomyocytes from oxidative stress–induced apoptosis by activating the PI3K/AKT pathway and inhibiting GSK3β [78]. Phosphorylation-mediated inactivation of GSK-3β suppresses BAX activation, thereby reducing apoptosis [69]. These protective effects may also be attributed to clusterin’s ability to inhibit BAX directly [79]. Furthermore, clusterin modulates oxidative stress and maintains mitochondrial integrity [80–83]. By regulating the aforementioned pathways, clusterin prevents apoptosis and enhances cell survival under stress [67, 84]. Given the correlation between upregulated clusterin expression and improved cardiovascular outcomes, clusterin can be regarded as a vital cardioprotective protein.

The aforementioned findings closely align with those of other experiments performed in this study. Among all identified DEGs, *Clu* exhibited the highest TPM values in cardiomyocytes treated with both the CM and DOX. The level of clusterin in HL-1 cells treated with DOX alone was approximately 30 ng/mL. By contrast, the levels of clusterin in DOX-exposed HL-1 cells treated with the CM and those treated with EVs were 127.2 ± 8.4 and 46.9 ± 5.5 ng/mL, respectively (Fig. 6E). Clusterin, a well-known stress-response protein [71], is crucial for cellular defense. Our findings suggest that CM or EV pretreatment enables HL-1 cells to exhibit a robust stress response to DOX treatment, supporting our hypothesis that EVS_ASC_ would play a protective or modulatory role in maintaining cellular resilience under stress conditions. The significant upregulation of clusterin expression indicates its pivotal role in mediating the cardioprotective effect of EVS_ASC_.

Clusterin knockdown significantly increased the ratio of cleaved caspase-3 to procaspase-3, highlighting clusterin as a crucial mediator of the antiapoptotic effect of EVS_ASC_. This conclusion is further substantiated by the observed increases in the levels of phosphorylated AKT [85], BAD [22], and GSK3β [78] in the DOX + CM group, highlighting key pathways involved in cardioprotection. Notably, the CM upregulated clusterin expression, even in untreated cardiomyocytes. The ability of the CM to mitigate apoptosis under DOX-induced stress conditions highlights its therapeutic potential grounded in its cytoprotective properties. Overall, EVS_ASC_ alleviates DOX-induced apoptosis in cardiomyocytes, at least partially, by upregulating the expression of clusterin. Future studies are required to delineate the differential regulation of clusterin under stressed versus nonstressed conditions and to evaluate its broader implications in cardioprotection. Our findings suggest that EVS_ASC_ mitigates DIC and that clusterin upregulation can serve as a biomarker of the efficacy of ASC-based therapies.

In our study, both the CM and EVs significantly upregulated clusterin expression (Fig. 6E) and counteracted DOX-induced reductions in viability (Fig. 6G) in HL-1 cardiomyocytes. However, considerable differences were observed between the two treatments: the level of clusterin secretion was substantially higher in CM-treated cells than in EV-treated cells (127.2 ± 8.4 vs. 46.9 ± 5.5 ng/mL). This finding suggests that the presence of additional soluble factors within the CM synergize to amplify clusterin production. Notably, EVs exerted a relatively targeted effect, with pronounced mitochondrial localization. Therefore, CM and EVs may exert effects through complementary but distinct cardioprotective mechanisms. These findings align with those of research highlighting the unique and overlapping roles of EVs and the broader secretome. For example, He et al. demonstrated that small EVs derived from adipose tissue and those derived from adipose tissue extract exhibit distinct biological signatures; the CM exerts a comprehensive therapeutic effect by combining EVs and soluble factors [86]. Additionally, Carceller et al. reported that the anti-inflammatory effect of the MSC secretome is not entirely mediated by EVs, highlighting independent contributions from soluble proteins, cytokines, and growth factors [87]. Giannasi et al. indicated that an ASC-derived secretome outperformed isolated EVs in an osteoarthritis model because of the inclusion of non-EV-associated proteins and metabolites [88]. Constantinou et al. stated that the protective effects of cardiac MSC-CM on hiPSC-derived cardiomyocytes under oxidative stress were primarily mediated by nonexosomal components [89]. In our study, the CM contained a spectrum of ASC-secreted bioactive compounds, such as soluble proteins, cytokines, and metabolites, in addition to EVs. This broader composition likely explains the CM’s superior ability to upregulate clusterin expression. However, this diversity of composition also presents challenges in terms of standardization and scalability. Conversely, EVs, with their well-defined molecular cargo, offer a targeted and scalable therapeutic option, although they may require supplementation with soluble factors to fully replicate the multifaceted effects of CM. In the present study, clusterin knockdown confirmed the importance of this protein as a mediator of cardioprotection. The loss of the protective effects of the CM and EVs after clusterin knockdown suggests that clusterin is not only a byproduct but also an essential functional component. Although CM treatment leads to higher levels of clusterin secretion than does EV treatment, EVs alone can exert clusterin-dependent antiapoptotic effects, offering a simple therapeutic platform with few extraneous variables. This balance between complexity and focus highlights the CM and EVs as complementary strategies for developing advanced cardioprotective therapies.

Our findings are consistent with those of He et al., who demonstrated that clusterin present in UC-MSC-derived EVs directly mediates the protective effects of these vesicles on ovarian granulosa cells; genetic silencing of clusterin blocked this effect [90]. Both the present and previous studies highlight clusterin as a key effector molecule in the cytoprotective role of MSC-derived EVs across tissues. However, a notable mechanistic difference is evident between the two studies. In He et al., EVs directly delivered clusterin to recipient cells, with this occurring through a paracrine mechanism. By contrast, in our study, EVs partially stimulated cardiomyocytes to upregulate clusterin expression, employing an indirect mechanism. This mechanistic difference raises the intriguing question of whether EVs function primarily by directly delivering cardioprotective proteins to recipient cells or by reprogramming endogenous pathways in these cells. The mechanism of action must be clarified to optimize EV-based therapeutic strategies, potentially by enhancing clusterin levels in EVs or by maximizing the ability of these vesicles to induce protective gene expression in target tissues. Future research should explore the intracellular signaling cascades through which EVs upregulate clusterin expression. Furthermore, future studies should determine the relative functional contributions of EV-delivered versus endogenously induced clusterin.

We observed that murine ASC-derived CM significantly counteracted DOX-induced reductions in the metabolic activity of hiPSC-derived cardiomyocytes. Moreover, the CM partially restored clusterin levels reduced by DOX treatment. These results underscore the conserved nature of key pathways that mediate the cardioprotective effects of paracrine factors across species, such as the PI3K/AKT pathway. Nonetheless, the translational applicability of murine ASC-derived CM requires careful consideration. Species-specific differences in the composition and activity of CM or EV cargo, such as miRNAs, proteins, and cytokines, may influence the extent of their therapeutic effects on human cells. Furthermore, when administered to human cardiomyocytes, murine ASC-derived factors may exhibit different immunogenic and compatibility profiles, which may alter their safety and efficacy. Functional analyses in human cardiomyocytes must be performed to assess the effects of DOX beyond apoptosis or mitochondrial ROS production, including its effects on the cells’ contractility and calcium handling capacity. Understanding how CM or EVs affect the aforementioned parameters and how clusterin mediates these effects would be highly beneficial. To address the limitations of this study and strengthen the translational relevance of our results, future studies should validate our findings by investigating the effects of human ASC-derived CM or EVs on hiPSC-derived cardiomyocytes [70]. For example, future research efforts can be invested in elucidating the molecular pathways underlying the downregulation of clusterin expression in hiPSC-derived cardiomyocytes and in determining how ASC-derived CM or EVs can prevent or reverse this downregulation. Such efforts would help confirm whether the cardioprotective effects observed in our study extend to human-derived systems, thereby paving the path for clinical applications.

This study has several limitations. First, our findings were obtained exclusively through in vitro experiments performed using mouse ASCs, which may limit the direct applicability of the results to humans. For the present study, we deliberately selected female FVB/N mice for ASC isolation to ensure consistency with our long-term research goals involving breast cancer–bearing mouse models. Second, pretreatment models are widely used to establish proof of concept for protective interventions, which can subsequently be adapted for clinical application. Future studies are required to expand our findings from pretreatment models to include cotreatment and posttreatment approaches. These approaches must be evaluated in vivo to better simulate clinical conditions and comprehensively assess of the therapeutic potential of EVS_ASC_. Third, the incorporation of FBS-derived components in CM preparation might have confounded the secretome-related results. Future studies may consider defined serum-free [91, 92] or chemically defined media to eliminate possible exogenous effects. Fourth, we did not explore the tumor-suppressing or tumor-promoting effects of clusterin, which emerged as a key driver of the effects of EVS_ASC_ on cardiomyocytes. Clusterin has been demonstrated to promote cancer cell survival, proliferation, and metastasis through signaling cascades such as adenosine monophosphate–activated protein kinase/mechanistic target of rapamycin/Unc-51 like autophagy activating kinase 1 [93] and AKT/GSK3β/β-catenin [94] pathways. Although secreted clusterin (sCLU) is commonly overexpressed in colon carcinoma [95], reduced levels of sCLU have been associated with poor prognoses in prostate cancers [96]. These contrasting findings highlight the unclear and potentially dual role of sCLU in carcinogenesis [97, 98]. Therefore, to clarify the cardioprotective versus oncogenic potential of clusterin, future research should investigate its expression patterns and functional effects on both EVS_ASC_-treated cardiomyocytes and cancer cells. Finally, we did not evaluate the oncogenic potential of EVs themselves [99, 100]. Small EVs derived from cardiac MSCs have been demonstrated to enhance tumor growth after myocardial infarction by delivering oncogenic cargo, including tumor-promoting cytokines, growth factors, and microRNAs [100]. In the present study, we used EVs derived from normal ASCs, which likely differ in content and biological function from cardiac MSC–derived small EVs. Nonetheless, the potential effect of ASC-derived EVs on tumor behavior warrants direct investigation. Future studies should characterize the tumor-related effects of ASC-derived EVs and develop strategies that maximize their therapeutic value while minimizing any potential risks related to cancer promotion.

## Conclusion

This study suggests that EVS_ASC_ significantly reduces apoptosis and caspase-3 activation in cardiomyocytes; this protective effect persists even after 24 h from DOX withdrawal. Our findings further confirm the intracellular localization and sustained presence of EVs under chemotherapeutic stress, underscoring their active involvement in cardioprotection. Among the key molecular mediators investigated, clusterin emerged as a pivotal effector: knocking down clusterin expression through relevant siRNA markedly diminished the cardioprotective effect of EVS_ASC_. This result highlights the essential role of clusterin in cardioprotection. Mechanistic data indicate that EVS_ASC_ activates AKT signaling, inhibits BAD and GSK3β, and reduces mitochondrial ROS production. Our findings may inform innovative strategies for improving treatment outcomes in cancer, particularly those aimed at alleviating drug-induced cardiotoxicity during chemotherapy.

## Electronic supplementary material

Below is the link to the electronic supplementary material.

Supplementary Material 1

Supplementary Material 2

Supplementary Material 3

Supplementary Material 4

## Data Availability

The datasets used and analyzed during this study are available from the corresponding author upon reasonable request. The sequencing data supporting the results are provided in Table S2.

## Abbreviations

ASCs: Adipose-derived stem cells
BM-MSCs: Bone marrow mesenchymal stromal cells
CCK-8: Cell Counting Kit-8
CM: Conditioned medium
DEGs: Differentially expressed genes
DIC: Doxorubicin-induced cardiotoxicity
DOX: Doxorubicin
DRZ: Dexrazoxane
ELISA: Enzyme-linked immunosorbent assay
EV: Extracellular vesicle
EVS: Extracellular vesicle-enriched secretome
EVS_ASC_: Extracellular vesicle-enriched secretome derived from adipose-derived stem cells
FBS: Fetal bovine serum
HGF: Hepatocyte growth factor
HCI: High-content imaging
IGF-1: Insulin-like growth factor
MSCs: Mesenchymal stem cells
mtSOX: Mitochondrial superoxide
OCR: Oxygen consumption rate
PBS: Phosphate-buffered saline
ROS: Reactive oxygen species
RNA-seq: RNA sequencing
sCLU: Secretory form of clusterin
TPM: Transcripts per million
UC-MSCs: Umbilical cord mesenchymal stromal cells
