# Supplementary material for: Extracellular vesicle-enriched secretome of adipose-derived stem cells upregulates clusterin to alleviate doxorubicin-induced apoptosis in cardiomyocytes
Source: Biol Direct. 2025 Jul 16;20:84. doi: 10.1186/s13062-025-00664-5 (PMC12265340; doi:10.1186/s13062-025-00664-5)
Supplement: Supplementary file 4 — Supplementary Material 4 [file 13062_2025_664_MOESM4_ESM.docx]

**Extracellular Vesicle-Enriched Secretome of Adipose-Derived Stem Cells Upregulates Clusterin to Alleviate Doxorubicin-Induced Apoptosis in Cardiomyocytes**

Wan-Tseng Hsu, PhD^1^**^*^**, Shinji Kobuchi, PhD^2^, Tung-Chun Chien, PharmD^3^, I-Chun Chen, MD, PhD^4,5,6^, Shohei Hamada, PhD^7^, Masayuki Tsujimoto, PhD^8^, I-Lin Tsai^9^, Yun-Sheng Wong^1^, Kuan-Hsuan Tung^1^, Ying-Zhen He^1^

^1,2^ School of Pharmacy, College of Medicine, National Taiwan University, Taipei, Taiwan;

^2^ Laboratory of Pharmacokinetics, Kyoto Pharmaceutical University, Kyoto, Japan;

^3^ Graduate Institute of Clinical Pharmacy, College of Medicine, National Taiwan University, Taipei, Taiwan;

^4^ Department of Medical Oncology, National Taiwan University Cancer Center, Taipei, Taiwan;

^5^ Department of Oncology, National Taiwan University Hospital, Taipei, Taiwan;

^6^ Graduate Institute of Oncology, College of Medicine, National Taiwan University, Taipei, Taiwan;

^7^ Laboratory of Pharmaceutical Chemistry, Kyoto Pharmaceutical University, Kyoto, Japan;

^8^ Laboratory of Clinical Pharmacy, Kyoto Pharmaceutical University, Kyoto, Japan;

^9^ Department of Biochemistry and Molecular Cell Biology, School of Medicine, College of Medicine, Taipei Medical University, Taipei, Taiwan.

**Supplementary Figures**


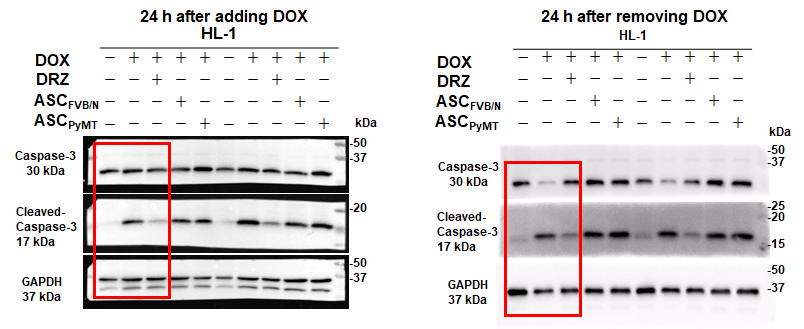


**Supplemental Figure 1.** **Corresponding uncropped full-length gels and blots in Figure 1D.** Red boxes indicated the cropped gels and blots in the main text.


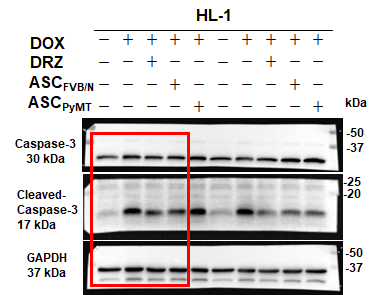


**Supplemental Figure 2. Corresponding uncropped full-length gels and blots in Figure 2D.** Red boxes indicated the cropped gels and blots in the main text.


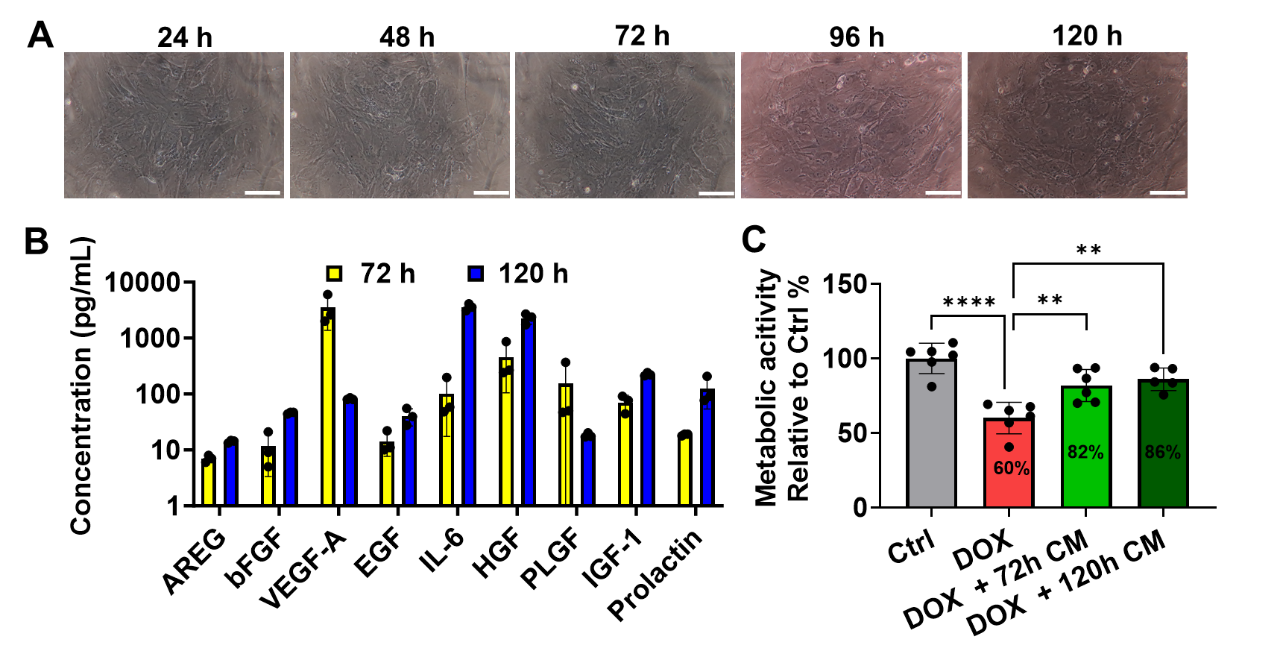


**Supplemental Figure 3. Detection of cardioprotective factors in conditioned medium from ASCs**

**A.** Collection of conditioned medium (CM) at 72 and 120 hours. **B.** Comparison of concentrations of cardioprotective factors between the two CM collection times. **C.** Evaluation of the protective effects of CM on doxorubicin-induced cardiotoxicity (DIC).


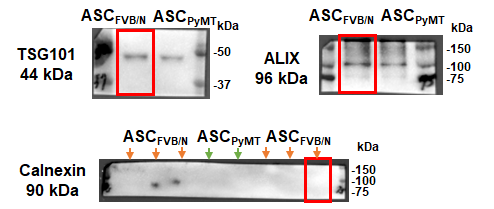


**Supplemental Figure 4.** **Corresponding uncropped full-length gels and blots in Figure 4F.** Red boxes indicated the cropped gels and blots in the main text.

**
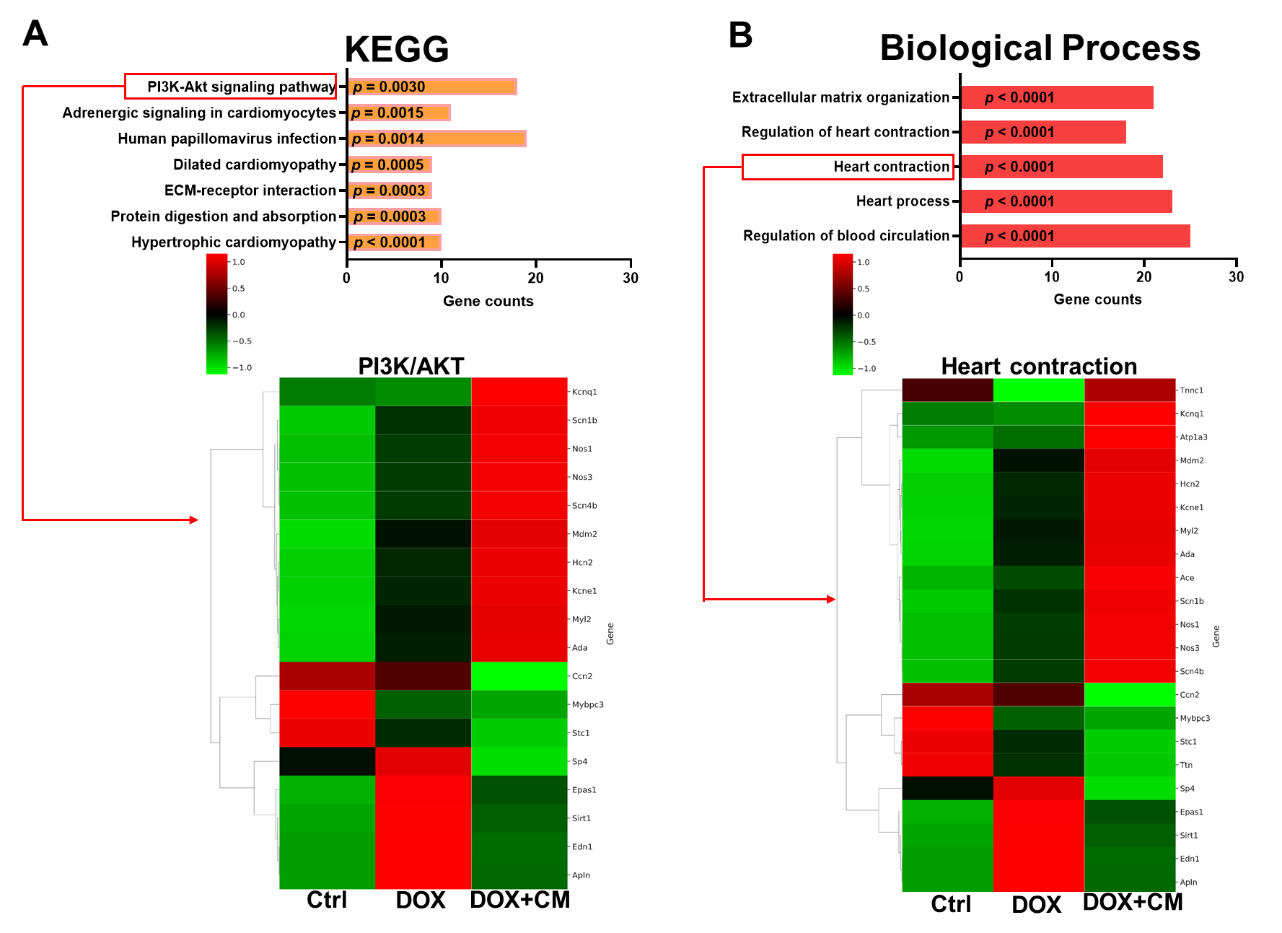
**

**Supplemental Figure 5. A.** KEGG pathway enrichment analysis and **B.** Biological Process Gene Ontology enrichment analysis of upregulated signaling pathways in the DOX-treated pretreated with conditioned medium (CM) group compared to the DOX-only treated group. The x-axis represents the percentage of significant genes contributing to each pathway. Notably, the PI3K/AKT signaling pathway demonstrates a high level of enrichment. DEGs show significant involvement in processes related to heart contraction, underscoring their potential role in cardioprotection.


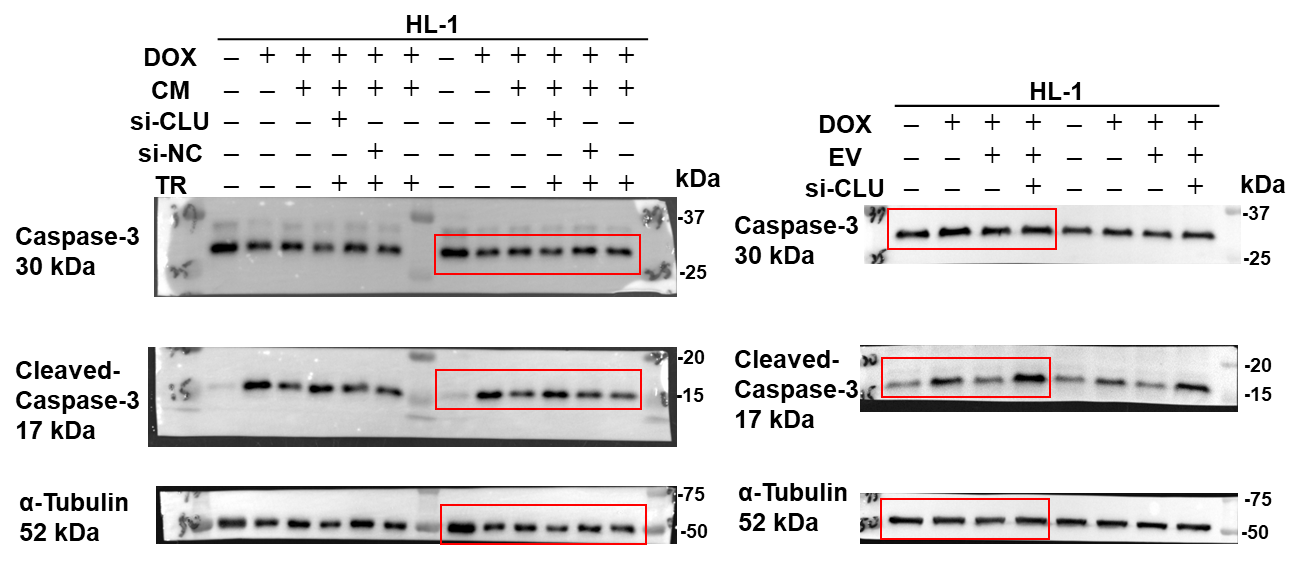


**Supplemental Figure 6. Corresponding uncropped full-length gels and blots in Figure 6H.** Red boxes indicated the cropped gels and blots in the main text.


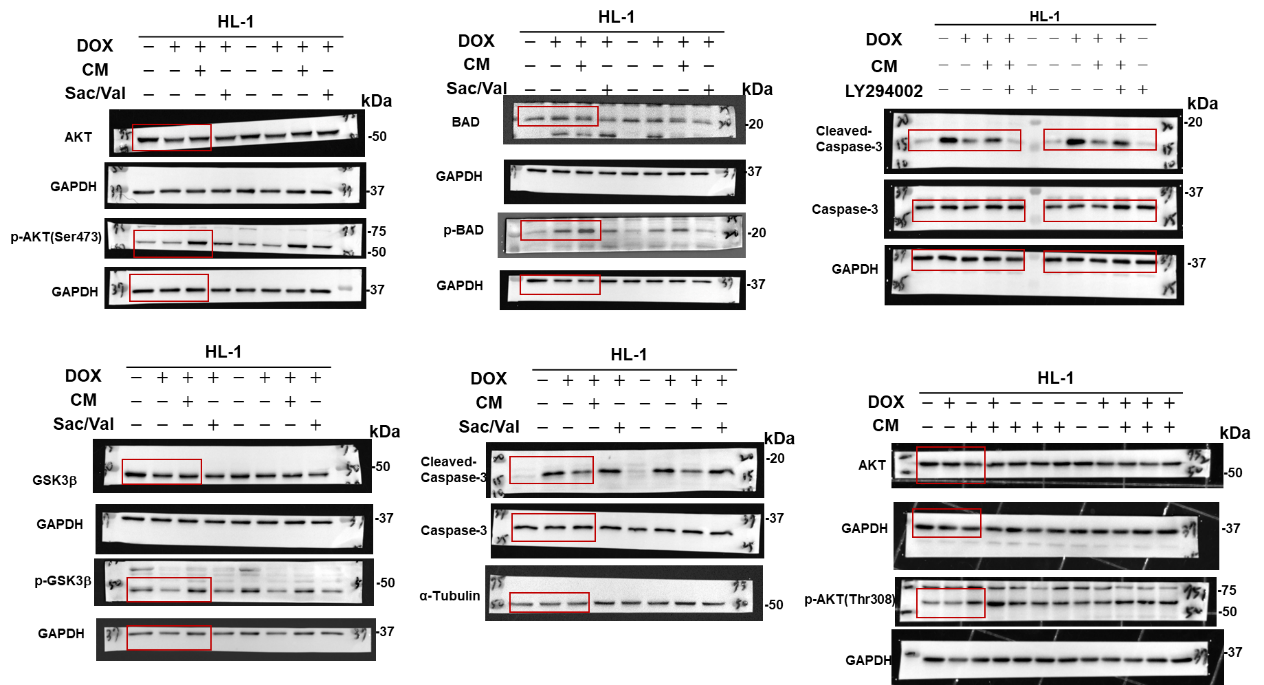


**Supplemental Figure 7. Corresponding uncropped full-length gels and blots in Figure 7A.** Red boxes indicated the cropped gels and blots in the main text.

**Supplementary Tables**

**Supplemental Table 1. Levels of cytokines (pg/mL) in the conditioned medium derived from ASCs were measured by using a Quantibody® Mouse Cytokine Array 4000 Kit.**

| **Factor name** | | **Mean** | | **SD** | | **Factor name** | | **Mean** | | **SD** | |
| --- | --- | --- | --- | --- | --- | --- | --- | --- | --- | --- | --- |
| 4-1BB | | 38.0 | | 33.3 | | CTLA-4 | | 0.0 | | 0.0 | |
| 6Ckine | | 1275.7 | | 609.7 | | CXCL15 | | 130169.0 | | 44424.2 | |
| ACE | | 452.0 | | 226.1 | | CXCL16 | | 207.7 | | 86.7 | |
| Activin A | | 11066.0 | | 6230.3 | | Cystatin C | | 3849.7 | | 1598.4 | |
| ADAMTS1 | | 254.0 | | 394.1 | | DAN | | 99.0 | | 36.3 | |
| Adiponectin | | 368.3 | | 470.9 | | Decorin | | 4983.7 | | 2975.1 | |
| ALK-1 | | 9.7 | | 7.6 | | Dkk-1 | | 2.3 | | 1.5 | |
| AR | | 7.0 | | 1.0 | | DLL4 | | 2959.7 | | 958.4 | |
| ANG-1 | | 26801.0 | | 22353.4 | | Dtk | | 37.3 | | 41.5 | |
| ANGPTL3 | | 7.7 | | 13.3 | | E-Cadherin | | 0.0 | | 0.0 | |
| Artemin | | 0.0 | | 0.0 | | EDAR | | 96.7 | | 95.0 | |
| Axl | | 1073.7 | | 713.9 | | EGF | | 14.3 | | 6.7 | |
| B7-1 | | 104.0 | | 29.5 | | Endocan | | 66.0 | | 34.7 | |
| BAFF R | | 123.3 | | 12.7 | | Endoglin | | 144.7 | | 70.1 | |
| bFGF | | 11.7 | | 8.3 | | Eotaxin | | 12.7 | | 4.2 | |
| BLC | | 0.0 | | 0.0 | | Eotaxin-2 | | 20.3 | | 10.1 | |
| BTC | | 2.3 | | 0.6 | | Epigen | | 445.7 | | 568.0 | |
| C5a | | 4.3 | | 2.3 | | Epiregulin | | 2.7 | | 4.6 | |
| CCL28 | | 0.0 | | 0.0 | | E-selectin | | 6.0 | | 10.4 | |
| CCL6 | | 10.0 | | 5.2 | | Fas | | 1.7 | | 0.6 | |
| CD27 | | 5350.7 | | 2810.6 | | Fas L | | 47.3 | | 3.1 | |
| CD27L | | 589.3 | | 193.8 | | Fcg RIIB | | 0.0 | | 0.0 | |
| CD30 | | 0.3 | | 0.6 | | Fetuin A | | 17394.3 | | 6924.3 | |
| CD30l | | 15.3 | | 4.2 | | Flt-3L | | 35.3 | | 18.0 | |
| CD36 | | 9.3 | | 16.2 | | Fractalkine | | 249.0 | | 78.6 | |
| CD40 | | 14.7 | | 3.5 | | Galectin-1 | | 6314.7 | | 3599.2 | |
| CD40L | | 109.0 | | 77.3 | | Galectin-3 | | 579.7 | | 301.7 | |
| CD48 | | 27.7 | | 5.0 | | Galectin-7 | | 352.7 | | 610.8 | |
| CD6 | | 10.3 | | 4.9 | | Gas 1 | | 1309.3 | | 685.8 | |
| Chemerin | | 1050.0 | | 478.9 | | Gas 6 | | 1391.7 | | 553.3 | |
| Chordin | | 689.0 | | 269.8 | | G-CSF | | 87.0 | | 31.6 | |
| Clusterin | | 14500.0 | | 8116.4 | | GITR | | 18.0 | | 7.0 | |
| CRP | | 0.0 | | 0.0 | | GITR L | | 2.3 | | 4.0 | |
| **Factor name** | **Mean** | | **SD** | | **Factor name** | | **Mean** | | **SD** | |  |
| CT-1 | 45.7 | | 40.1 | | GM-CSF | | 0.0 | | 0.0 | |  |
| gp130 | 2.7 | | 2.3 | | IL-23 | | 1301.0 | | 198.0 | |  |
| Granzyme B | 10.7 | | 9.3 | | IL-28 | | 13.7 | | 5.7 | |  |
| Gremlin | 440.3 | | 688.9 | | IL-3 | | 0.0 | | 0.0 | |  |
| H60 | 13.7 | | 7.2 | | IL-3 Rβ | | 2849.7 | | 1309.0 | |  |
| HAI-1 | 21.0 | | 12.3 | | IL-33 | | 64.3 | | 30.9 | |  |
| HGF | 456.3 | | 351.5 | | IL-4 | | 0.0 | | 0.0 | |  |
| HGF R | 129.3 | | 67.4 | | IL-5 | | 0.0 | | 0.0 | |  |
| ICAM-1 | 0.0 | | 0.0 | | IL-6 | | 101.3 | | 83.9 | |  |
| IFN-γ | 0.0 | | 0.0 | | IL-7 | | 133.7 | | 65.3 | |  |
| IFN-γ R1 | 0.0 | | 0.0 | | IL-7 Rα | | 1339.7 | | 499.5 | |  |
| IGFBP-2 | 11.3 | | 6.1 | | IL-9 | | 121.3 | | 143.0 | |  |
| IGFBP-3 | 1554.0 | | 1275.5 | | I-TAC | | 89.3 | | 26.6 | |  |
| IGFBP-5 | 484.7 | | 174.4 | | JAM-A | | 237.7 | | 130.3 | |  |
| IGFBP-6 | 6330.7 | | 2718.1 | | KC | | 95.3 | | 75.4 | |  |
| IGF-I | 70.7 | | 24.1 | | Kremen-1 | | 194.3 | | 10.7 | |  |
| IL-1 R4 | 283.7 | | 156.8 | | Leptin | | 35.7 | | 61.8 | |  |
| IL-10 | 10.7 | | 6.4 | | Leptin R | | 68.3 | | 32.5 | |  |
| IL-12p40 | 36.7 | | 4.0 | | Limitin | | 6.7 | | 2.9 | |  |
| IL-12p70 | 84.3 | | 29.8 | | Lipocalin-2 | | 13.7 | | 4.2 | |  |
| IL-13 | 38.3 | | 66.4 | | LIX | | 1.7 | | 2.9 | |  |
| IL-15 | 4868.7 | | 2445.7 | | LOX-1 | | 461.7 | | 452.9 | |  |
| IL-17 | 19.0 | | 11.3 | | L-Selectin | | 3.0 | | 5.2 | |  |
| IL-17B | 2862.7 | | 4689.8 | | MadCAM-1 | | 219.0 | | 106.9 | |  |
| IL-17B R | 4422.0 | | 818.8 | | Marapsin | | 497.0 | | 208.4 | |  |
| IL-17E | 121.0 | | 64.6 | | MBL-2 | | 6.0 | | 2.6 | |  |
| IL-17F | 137.0 | | 61.9 | | MCP-1 | | 0.0 | | 0.0 | |  |
| IL-1α | 0.0 | | 0.0 | | MCP-5 | | 7.7 | | 4.2 | |  |
| IL-1β | 3.7 | | 6.4 | | M-CSF | | 5.3 | | 4.0 | |  |
| IL-1rα | 58.3 | | 4.9 | | MDC | | 3.0 | | 1.0 | |  |
| IL-2 | 83.3 | | 144.3 | | Meteorin | | 37.3 | | 19.7 | |  |
| IL-2 Rα | 12.3 | | 5.1 | | MFG-E8 | | 17315.7 | | 12876.7 | |  |
| IL-20 | 133.0 | | 44.3 | | MIG | | 73.0 | | 35.8 | |  |
| IL-21 | 6.3 | | 11.0 | | MIP-1α | | 28.0 | | 10.4 | |  |
| IL-22 | 0.0 | | 0.0 | | MIP-1β | | 0.0 | | 0.0 | |  |

| **Factor name** | **Mean** | **SD** | **Factor name** | **Mean** | **SD** |
| --- | --- | --- | --- | --- | --- |
| MIP-1γ | 743.7 | 755.2 | SLAM | 187.3 | 324.5 |
| MIP-2 | 8.3 | 3.5 | TACI | 11.7 | 10.4 |
| MIP-3α | 12.3 | 3.5 | TARC | 38.3 | 14.6 |
| MIP-3β | 8.3 | 3.8 | TCA-3 | 0.0 | 0.0 |
| MMP-10 | 0.0 | 0.0 | TCK-1 | 0.0 | 0.0 |
| MMP-2 | 9620.3 | 4882.0 | TECK | 91.0 | 157.6 |
| MMP-3 | 9586.0 | 7783.6 | Testican 3 | 270.7 | 46.7 |
| Neprilysin | 348.3 | 162.2 | TGF-β1 | 8401.7 | 3781.2 |
| Nope | 545.7 | 282.0 | Thrombopoietin | 13987.0 | 1188.9 |
| NOV(CCN3) | 5243.0 | 4338.1 | TIM-1 | 254.7 | 138.8 |
| Osteoactivin | 407.7 | 217.1 | TNF RI | 715.3 | 405.9 |
| Osteopontin | 18205.0 | 5620.2 | TNF RII | 345.7 | 256.7 |
| Osteoprotegerin | 17057.7 | 6488.3 | TNFα | 19.7 | 7.6 |
| OX40 Ligand | 44.7 | 20.7 | TRAIL | 496.3 | 349.1 |
| P-Cadherin | 618.7 | 336.6 | TRANCE | 63.3 | 95.0 |
| PDGF-AA | 133.7 | 32.7 | TREM-1 | 9.7 | 9.1 |
| Pentraxin 3 | 838.7 | 903.8 | TremL1 | 371.3 | 254.1 |
| Periostin | 25589.7 | 13271.3 | TROY | 3.0 | 5.2 |
| Persephin | 0.0 | 0.0 | Tryptase ε | 52.3 | 24.9 |
| PF-4 | 0.0 | 0.0 | TSLP | 24.3 | 10.7 |
| PIGF-2 | 154.7 | 183.9 | TWEAK | 110.0 | 95.3 |
| Progranulin | 10588.3 | 10082.9 | TWEAK R | 80701.7 | 38651.5 |
| Prolactin | 124.7 | 70.8 | VCAM-1 | 661.7 | 427.0 |
| Pro-MMP-9 | 1983.3 | 411.4 | VEGF | 3542.7 | 2167.7 |
| Prostasin | 301.3 | 396.9 | VEGF R1 | 134.3 | 56.1 |
| P-selectin | 32.3 | 14.4 | VEGF R3 | 119.3 | 73.0 |
| RAGE | 36.7 | 12.7 | VEGF-B | 32.3 | 56.0 |
| RANTES | 252.3 | 134.9 | VEGF-D | 1.7 | 0.6 |
| Renin 1 | 2124.7 | 777.1 | VEGF-R2 | 1.3 | 1.5 |
| Resistin | 4.3 | 0.6 |  |  |  |
| SCF | 34.0 | 22.7 |  |  |  |
| SDF-1α | 1146.0 | 191.5 |  |  |  |
| sFRP-3 | 71894.7 | 37230.7 |  |  |  |
| Shh-N | 8.7 | 7.6 |  |  |  |

**Abbreviations:**

4-1BB: Tumor necrosis factor receptor superfamily member 9

6Ckine: C-C motif chemokine ligand 21 (CCL21)

ACE: Angiotensin-converting enzyme

Activin A: A member of the TGF-beta family of proteins

ADAMTS1: A disintegrin and metalloproteinase with thrombospondin motifs 1

Adiponectin: Adipocyte complement-related protein

ALK-1: Activin receptor-like kinase 1

AR: Amphiregulin

ANG-1: Angiopoietin-1

ANGPTL3: Angiopoietin-like protein 3

Artemin: A member of the glial cell line-derived neurotrophic factor (GDNF) family

Axl: A receptor tyrosine kinase

BAFF R: B-cell activating factor receptor

bFGF: Basic fibroblast growth factor

BLC: B-lymphocyte chemoattractant (CXCL13)

BTC: Betacellulin

C5a: Complement component 5a

CCL28: C-C motif chemokine ligand 28

CCL6: C-C motif chemokine ligand 6

CD27: Tumor necrosis factor receptor superfamily member 7

CD27L: CD70 (CD27 ligand)

CD30L: CD153 (CD30 ligand)

CD30: Tumor necrosis factor receptor superfamily member 8

CD36: Cluster of differentiation 36

CD40: Tumor necrosis factor receptor superfamily member 5

CD40L: CD154 (CD40 ligand)

CD48: Cluster of differentiation 48

CD6: Cluster of differentiation 6

CD80: B7-1 (T-lymphocyte activation antigen CD80)

Chemerin: Retinoic acid receptor responder protein 2

Chordin: A BMP antagonist

Clusterin: Apolipoprotein J

CRP: C-reactive protein

CT-1: Cardiotrophin-1

CTLA-4: Cytotoxic T-lymphocyte-associated protein 4

CXCL15: Chemokine (C-X-C motif) ligand 15

CXCL16: Chemokine (C-X-C motif) ligand 16

Cystatin C: A cysteine protease inhibitor

DAN: Differential screening-selected gene aberrant in neuroblastoma

Decorin: A proteoglycan associated with collagen

Dkk-1: Dickkopf-related protein 1

DLL4: Delta-like canonical Notch ligand 4

Dtk: Tyrosine-protein kinase receptor TYRO3

E-Cadherin: Epithelial cadherin

EDAR: Ectodysplasin A receptor

EGF: Epidermal growth factor

Endocan: Endothelial cell-specific molecule-1

Endoglin: A TGF-beta-binding protein

Eotaxin: C-C motif chemokine ligand 11 (CCL11)

Eotaxin-2: C-C motif chemokine ligand 24 (CCL24)

Epigen: Epigen growth factor

Epiregulin: Epidermal growth factor receptor ligand

E-selectin: Endothelial selectin

Fas: Fas receptor (CD95)

Fas L: Fas ligand

Fcg RIIB: Fc-gamma receptor IIb (CD32a)

Fetuin A: Alpha-2-HS-glycoprotein

Flt-3L: Fms-related tyrosine kinase 3 ligand

Fractalkine: CX3CL1

Galectin-1: Beta-galactoside-binding protein

Galectin-3: Lectin, galactoside-binding soluble 3

Galectin-7: Lectin, galactoside-binding soluble 7

Gas 1: Growth arrest-specific gene 1

Gas 6: Growth arrest-specific gene 6

G-CSF: Granulocyte colony-stimulating factor

GITR: Glucocorticoid-induced TNFR-related protein

GITR L: Glucocorticoid-induced TNFR-related protein ligand

GM-CSF: Granulocyte-macrophage colony-stimulating factor

gp130: Glycoprotein 130

Granzyme B: A serine protease found in cytotoxic T cells

Gremlin-1: A BMP antagonist

H60: Histocompatibility antigen 60

HAI-1: Hepatocyte growth factor activator inhibitor type 1

HGF: Hepatocyte growth factor

HGF R: Hepatocyte growth factor receptor (c-Met)

ICAM-1: Intercellular adhesion molecule 1

IFN-γ: Interferon gamma

IFN-γ R1: Interferon-gamma receptor 1

IGFBP-2: Insulin-like growth factor-binding protein 2

IGFBP-3: Insulin-like growth factor-binding protein 3

IGFBP-5: Insulin-like growth factor-binding protein 5

IGFBP-6: Insulin-like growth factor-binding protein 6

IGF-I: Insulin-like growth factor I

IL-1 R4: Interleukin-1 receptor 4 (ST2)

IL-10: Interleukin 10

IL-12p40: Interleukin-12 subunit p40

IL-12p70: Interleukin-12 subunit p70

IL-13: Interleukin 13

IL-15: Interleukin 15

IL-17: Interleukin 17

IL-17B: Interleukin 17B

IL-17B R: Interleukin 17B receptor

IL-17E: Interleukin 25

IL-17F: Interleukin 17F

IL-1α: Interleukin 1 alpha

IL-1β: Interleukin 1 beta

IL-1rα: Interleukin 1 receptor antagonist

IL-2: Interleukin 2

IL-2 Rα: Interleukin 2 receptor alpha

IL-20: Interleukin 20

IL-21: Interleukin 21

IL-22: Interleukin 22

IL-23: Interleukin 23

IL-28: Interleukin 28

IL-3: Interleukin 3

IL-3 Rβ: Interleukin 3 receptor beta

IL-33: Interleukin 33

IL-4: Interleukin 4

IL-5: Interleukin 5

IL-6: Interleukin 6

IL-7: Interleukin 7

IL-7 Rα: Interleukin 7 receptor alpha

IL-9: Interleukin 9

I-TAC: Interferon-inducible T-cell alpha chemoattractant (CXCL11)

JAM-A: Junctional adhesion molecule A

KC: Keratinocyte chemoattractant (CXCL1)

Kremen-1: Kringle-containing transmembrane protein 1

Leptin: Hormone regulating energy balance

Leptin R: Leptin receptor

Limitin: Alpha interferon-like cytokine

Lipocalin-2: Neutrophil gelatinase-associated lipocalin

LIX: Lipopolysaccharide-induced CXC chemokine

LOX-1: Lectin-like oxidized LDL receptor 1

L-Selectin: Leukocyte selectin (CD62L)

MadCAM-1: Mucosal vascular addressin cell adhesion molecule 1

Marapsin: A serine protease

MBL-2: Mannose-binding lectin 2

MCP-1: Monocyte chemoattractant protein 1 (CCL2)

MCP-5: Monocyte chemoattractant protein 5 (CCL12)

M-CSF: Macrophage colony-stimulating factor

MDC: Macrophage-derived chemokine (CCL22)

Meteorin: A neurotrophic factor

MFG-E8: Milk fat globule-EGF factor 8 protein

MIG: Monokine induced by gamma interferon (CXCL9)

MIP-1α: Macrophage inflammatory protein 1 alpha (CCL3)

MIP-1β: Macrophage inflammatory protein 1 beta (CCL4)

MIP-1γ: Macrophage inflammatory protein 1 gamma

MIP-2: Macrophage inflammatory protein 2 (CXCL2)

MIP-3α: Macrophage inflammatory protein 3 alpha (CCL20)

MIP-3β: Macrophage inflammatory protein 3 beta (CCL19)

MMP-10: Matrix metallopeptidase 10

MMP-2: Matrix metallopeptidase 2

MMP-3: Matrix metallopeptidase 3

Neprilysin: Neutral endopeptidase (NEP)

Nope: Neuralized-like E3 ubiquitin-protein ligase 4 (NEURL4)

NOV (CCN3): Nephroblastoma overexpressed gene

Osteoactivin: Glycoprotein nonmetastatic melanoma protein B (GPNMB)

Osteopontin: Secreted phosphoprotein 1 (SPP1)

Osteoprotegerin: Tumor necrosis factor receptor superfamily member 11b

OX40 Ligand: TNFSF4 (Tumor necrosis factor ligand superfamily member 4)

P-Cadherin: Placental cadherin

PDGF-AA: Platelet-derived growth factor subunit A

Pentraxin 3: Long pentraxin 3

Periostin: Osteoblast-specific factor 2 (OSF-2)

Persephin: A member of the GDNF family

PF-4: Platelet factor 4 (CXCL4)

PIGF-2: Placental growth factor-2

Progranulin: Granulin-epithelin precursor

Prolactin: A hormone associated with lactation

Pro-MMP-9: Pro-matrix metallopeptidase 9

Prostasin: Serine protease 8

P-selectin: Platelet selectin (CD62P)

RAGE: Receptor for advanced glycation end-products

RANTES: Regulated on activation, normal T cell expressed and secreted (CCL5)

Renin 1: Renin enzyme precursor

Resistin: Adipocyte-specific secretory factor

SCF: Stem cell factor (KIT ligand)

SDF-1α: Stromal cell-derived factor 1 alpha (CXCL12)

sFRP-3: Secreted frizzled-related protein 3

Shh-N: Sonic hedgehog N-terminal signaling domain

SLAM: Signaling lymphocytic activation molecule

TACI: Transmembrane activator and CAML interactor (TNFRSF13B)

TARC: Thymus and activation-regulated chemokine (CCL17)

TCA-3: T-cell activation gene 3 (CCL1)

TCK-1: Thymocyte chemoattractant 1

TECK: Thymus-expressed chemokine (CCL25)

Testican 3: Proteoglycan 3

TGF-β1: Transforming growth factor beta 1

Thrombopoietin: A regulator of platelet production

TIM-1: T-cell immunoglobulin and mucin domain 1

TNF RI: Tumor necrosis factor receptor I

TNF RII: Tumor necrosis factor receptor II

TNFα: Tumor necrosis factor alpha

TRAIL: TNF-related apoptosis-inducing ligand

TRANCE: TNF-related activation-induced cytokine (RANKL)

TREM-1: Triggering receptor expressed on myeloid cells 1

TremL1: Triggering receptor expressed on myeloid cells-like 1

TROY: Tumor necrosis factor receptor superfamily member 19

Tryptase ε: Tryptase epsilon

TSLP: Thymic stromal lymphopoietin

TWEAK: TNF-like weak inducer of apoptosis

TWEAK R: TWEAK receptor (FN14)

VCAM-1: Vascular cell adhesion molecule 1

VEGF: Vascular endothelial growth factor A

VEGF R1: Vascular endothelial growth factor receptor 1 (FLT1)

VEGF R3: Vascular endothelial growth factor receptor 3 (FLT4)

VEGF-B: Vascular endothelial growth factor B

VEGF-D: Vascular endothelial growth factor D

VEGF-R2: Vascular endothelial growth factor receptor 2 (KDR/FLK1)

**Table S2. List of the 126 protein-coding differentially expressed genes, based on the CM-to-DOX ratio in the transcripts per million (TPM) values**

| **Gene_ID** | **Gene name** | **Ctrl_TPM** | **DOX_TPM** | **CM_TPM** |
| --- | --- | --- | --- | --- |
|  |  |  |  |  |
| ENSMUSG00000022037 | Clu | 362.4838 | 431.4523 | 1286.5205 |
| ENSMUSG00000021702 | Thbs4 | 264.4316 | 172.2982 | 476.2258 |
| ENSMUSG00000030218 | Mgp | 241.5851 | 148.2940 | 366.3309 |
| ENSMUSG00000001025 | S100a6 | 141.8232 | 131.1899 | 304.8254 |
| ENSMUSG00000034361 | Cpne2 | 63.4752 | 86.4900 | 184.3788 |
| ENSMUSG00000091898 | Tnnc1 | 146.3821 | 78.2520 | 166.9961 |
| ENSMUSG00000029017 | Pmpcb | 162.1082 | 67.0549 | 158.0095 |
| ENSMUSG00000039396 | Neil3 | 187.2042 | 341.1743 | 157.1936 |
| ENSMUSG00000025153 | Fasn | 187.1711 | 330.1367 | 155.4694 |
| ENSMUSG00000028885 | Smpdl3b | 36.9967 | 61.7010 | 128.5042 |
| ENSMUSG00000059714 | Flot1 | 145.5604 | 62.9438 | 127.4730 |
| ENSMUSG00000055817 | Mta3 | 38.8993 | 41.9330 | 87.8082 |
| ENSMUSG00000003500 | Impdh1 | 30.0903 | 35.2220 | 72.9348 |
| ENSMUSG00000020808 | Pimreg | 127.4415 | 153.6320 | 70.9824 |
| ENSMUSG00000025083 | Afap1l2 | 16.2751 | 32.7634 | 69.4790 |
| ENSMUSG00000024854 | Pold4 | 24.2400 | 32.3447 | 66.1625 |
| ENSMUSG00000020644 | Id2 | 75.7197 | 148.4997 | 65.9364 |
| ENSMUSG00000044934 | Zfp367 | 73.9897 | 123.2309 | 61.5915 |
| ENSMUSG00000029763 | Exoc4 | 89.8628 | 29.9707 | 60.4474 |
| ENSMUSG00000002804 | Nudt14 | 22.8319 | 28.7570 | 57.5628 |
| ENSMUSG00000052031 | Tagap1 | 59.5743 | 121.1235 | 57.4315 |
| ENSMUSG00000032531 | Amotl2 | 74.6593 | 113.2790 | 53.9869 |
| ENSMUSG00000038775 | Vill | 28.5133 | 25.4925 | 53.0936 |
| ENSMUSG00000029201 | Ugdh | 107.2722 | 114.3549 | 52.9500 |
| ENSMUSG00000024843 | Chka | 30.0443 | 121.9141 | 50.9217 |
| ENSMUSG00000022555 | Dgat1 | 29.0417 | 106.6220 | 46.6694 |
| ENSMUSG00000038400 | Pmepa1 | 20.2155 | 19.7432 | 44.0175 |
| ENSMUSG00000026605 | Cenpf | 115.1792 | 101.7283 | 43.9441 |
| ENSMUSG00000116165 | Pdxp | 49.0252 | 93.1341 | 43.0559 |
| ENSMUSG00000042903 | Foxo4 | 64.4180 | 89.7160 | 41.8009 |
| ENSMUSG00000042515 | Pwwp3b | 63.9154 | 89.3187 | 40.5524 |
| ENSMUSG00000030037 | Mrpl53 | 44.3683 | 18.0608 | 39.6331 |
| ENSMUSG00000026245 | Farsb | 73.2355 | 16.2174 | 38.6317 |
| ENSMUSG00000021485 | Mxd3 | 42.2733 | 89.4462 | 37.8288 |
| **Gene_ID** | **Gene name** | **Ctrl_TPM** | **DOX_TPM** | **CM_TPM** |
| ENSMUSG00000051890 | Klhdc1 | 7.9704 | 14.8457 | 36.5297 |
| ENSMUSG00000041453 | Rpl21 | 33.9183 | 8.8032 | 36.0608 |
| ENSMUSG00000022034 | Esco2 | 51.7745 | 79.4867 | 34.7883 |
| ENSMUSG00000069135 | Fgfr1op | 33.0141 | 69.8645 | 33.6491 |
| ENSMUSG00000039795 | Zfand1 | 11.2698 | 11.2635 | 33.0044 |
| ENSMUSG00000014905 | Dnajb9 | 38.9756 | 66.2198 | 32.9419 |
| ENSMUSG00000024500 | Ppp2r2b | 17.2444 | 14.0252 | 31.9507 |
| ENSMUSG00000118504 | 4933434E20Rik | 21.1243 | 67.4973 | 31.5767 |
| ENSMUSG00000022096 | Hr | 31.8730 | 14.6177 | 31.3639 |
| ENSMUSG00000029385 | Ccng2 | 36.7850 | 83.8107 | 30.9981 |
| ENSMUSG00000030337 | Vamp1 | 13.9979 | 69.2690 | 30.9483 |
| ENSMUSG00000046056 | Sbsn | 9.0353 | 5.5263 | 29.8679 |
| ENSMUSG00000026042 | Col5a2 | 27.2634 | 13.5649 | 29.8396 |
| ENSMUSG00000044734 | Serpinb1a | 7.7647 | 8.6094 | 29.0984 |
| ENSMUSG00000045414 | Dipk2a | 105.0958 | 63.4996 | 28.7250 |
| ENSMUSG00000046027 | Stard5 | 7.5679 | 12.8354 | 27.6881 |
| ENSMUSG00000024298 | Zfp871 | 44.1803 | 57.1961 | 27.2294 |
| ENSMUSG00000016458 | Wt1 | 9.8027 | 11.5480 | 27.1026 |
| ENSMUSG00000068762 | Gstm6 | 7.6476 | 12.4385 | 26.4294 |
| ENSMUSG00000027326 | Knl1 | 44.8539 | 54.8783 | 26.3898 |
| ENSMUSG00000046295 | Ankle1 | 16.6274 | 74.3548 | 26.3871 |
| ENSMUSG00000007872 | Id3 | 15.1971 | 69.9550 | 23.7572 |
| ENSMUSG00000028175 | Depdc1a | 64.5006 | 47.6627 | 23.4839 |
| ENSMUSG00000027104 | Atf2 | 25.2608 | 53.5683 | 23.4389 |
| ENSMUSG00000020063 | Sirt1 | 15.6731 | 61.7554 | 23.3856 |
| ENSMUSG00000043183 | Simc1 | 13.9839 | 46.2692 | 22.4996 |
| ENSMUSG00000030681 | Mvp | 9.6611 | 8.2519 | 22.0913 |
| ENSMUSG00000036902 | Neto2 | 37.2376 | 50.4713 | 21.8833 |
| ENSMUSG00000060510 | Zfp266 | 44.2904 | 43.9380 | 21.7469 |
| ENSMUSG00000040152 | Thbs1 | 13.1674 | 46.2633 | 21.5480 |
| ENSMUSG00000026730 | Pter | 12.4860 | 10.0595 | 21.3793 |
| ENSMUSG00000000078 | Klf6 | 19.9728 | 53.6335 | 19.6699 |
| ENSMUSG00000048249 | Crebrf | 24.4400 | 43.5391 | 19.5617 |
| ENSMUSG00000015659 | Serac1 | 24.6064 | 41.5693 | 19.4496 |
| ENSMUSG00000005949 | Ctns | 25.4455 | 41.3542 | 19.2370 |
| ENSMUSG00000052854 | Nrk | 24.1862 | 44.2606 | 18.9606 |
| ENSMUSG00000019997 | Ccn2 | 48.2997 | 41.9889 | 18.9172 |
| **Gene_ID** | **Gene name** | **Ctrl_TPM** | **DOX_TPM** | **CM_TPM** |
| ENSMUSG00000034765 | Dusp5 | 13.1013 | 41.0619 | 17.7363 |
| ENSMUSG00000036377 | C530008M17Rik | 66.0934 | 37.8402 | 17.6266 |
| ENSMUSG00000029359 | Tesc | 8.6383 | 6.2795 | 17.0925 |
| ENSMUSG00000092035 | Peg10 | 16.4956 | 40.9242 | 17.0624 |
| ENSMUSG00000035967 | Ints6l | 19.4252 | 34.3125 | 16.9352 |
| ENSMUSG00000024378 | Stard4 | 41.4468 | 34.1867 | 16.5649 |
| ENSMUSG00000022773 | Ypel1 | 13.1015 | 51.2686 | 16.4029 |
| ENSMUSG00000025037 | Maoa | 31.9140 | 33.5183 | 16.2017 |
| ENSMUSG00000062901 | Klhl24 | 49.8873 | 38.4072 | 16.1737 |
| ENSMUSG00000025323 | Sp4 | 23.1812 | 32.1343 | 15.6347 |
| ENSMUSG00000053897 | Slc39a8 | 15.0794 | 30.9547 | 14.7869 |
| ENSMUSG00000024750 | Zfand5 | 26.8782 | 31.2740 | 14.6878 |
| ENSMUSG00000009545 | Kcnq1 | 6.1391 | 5.8760 | 14.4341 |
| ENSMUSG00000047446 | Arl4a | 24.0445 | 29.1230 | 14.0284 |
| ENSMUSG00000046785 | Epm2aip1 | 15.6073 | 28.9976 | 13.6198 |
| ENSMUSG00000047604 | Frat2 | 6.9934 | 32.2357 | 13.5123 |
| ENSMUSG00000032009 | Sesn3 | 17.4752 | 27.2774 | 13.4132 |
| ENSMUSG00000026094 | Stk17b | 20.9729 | 28.0578 | 13.0647 |
| ENSMUSG00000018983 | E2f2 | 16.3143 | 29.0261 | 13.0315 |
| ENSMUSG00000048450 | Msx1 | 7.7689 | 27.4111 | 12.8627 |
| ENSMUSG00000021835 | Bmp4 | 31.0663 | 30.4437 | 12.3420 |
| ENSMUSG00000038065 | Mturn | 15.1509 | 25.7933 | 12.0453 |
| ENSMUSG00000034422 | Parp14 | 6.5942 | 29.3967 | 11.9852 |
| ENSMUSG00000020653 | Klf11 | 15.2899 | 24.1664 | 11.7702 |
| ENSMUSG00000071042 | Rasgrp3 | 13.2118 | 26.3687 | 11.6844 |
| ENSMUSG00000056025 | Clca3a1 | 8.4981 | 5.7008 | 11.5491 |
| ENSMUSG00000057706 | Mex3b | 13.8713 | 27.7600 | 11.2374 |
| ENSMUSG00000026196 | Bard1 | 25.7513 | 26.7983 | 11.1196 |
| ENSMUSG00000031994 | Adamts8 | 12.4063 | 27.2359 | 10.8336 |
| ENSMUSG00000033949 | Trim36 | 8.6535 | 22.0904 | 10.6971 |
| ENSMUSG00000044390 | Tigd3 | 9.2795 | 23.5051 | 10.5541 |
| ENSMUSG00000045932 | Ifit2 | 35.6804 | 24.6953 | 9.9497 |
| ENSMUSG00000026235 | Epha4 | 26.4679 | 23.9537 | 9.8423 |
| ENSMUSG00000036882 | Arhgap33 | 30.3848 | 27.4291 | 9.8416 |
| ENSMUSG00000059674 | Cdh24 | 13.6320 | 22.3871 | 9.5409 |
| ENSMUSG00000024140 | Epas1 | 6.2655 | 19.9314 | 9.2927 |
| ENSMUSG00000022893 | Adamts1 | 15.5090 | 18.7876 | 8.8553 |
| **Gene_ID** | **Gene name** | **Ctrl_TPM** | **DOX_TPM** | **CM_TPM** |
| ENSMUSG00000028341 | Nr4a3 | 7.1321 | 18.3594 | 8.7045 |
| ENSMUSG00000098374 | Gm28043 | 23.0202 | 20.5157 | 8.3870 |
| ENSMUSG00000051650 | B3gnt2 | 19.7494 | 32.6979 | 8.0392 |
| ENSMUSG00000032446 | Eomes | 9.2336 | 17.1102 | 7.8709 |
| ENSMUSG00000078185 | Chml | 8.9667 | 18.5539 | 7.6875 |
| ENSMUSG00000049791 | Fzd4 | 14.1502 | 15.4271 | 7.6755 |
| ENSMUSG00000027796 | Smad9 | 18.5676 | 19.4134 | 7.5670 |
| ENSMUSG00000021367 | Edn1 | 5.6156 | 19.7524 | 7.3322 |
| ENSMUSG00000025612 | Bach1 | 6.5048 | 14.6466 | 7.2367 |
| ENSMUSG00000032724 | Abtb2 | 8.5809 | 14.3096 | 6.8837 |
| ENSMUSG00000107023 | Gm42715 | 13.3588 | 16.9094 | 6.5752 |
| ENSMUSG00000030680 | Pagr1a | 13.3154 | 18.9124 | 6.3400 |
| ENSMUSG00000050786 | Ccdc126 | 18.8605 | 13.7117 | 6.1242 |
| ENSMUSG00000027784 | Ppm1l | 17.3199 | 13.6010 | 6.0735 |
| ENSMUSG00000047344 | Lancl3 | 10.3073 | 12.2216 | 5.6890 |
| ENSMUSG00000074505 | Fat3 | 10.7596 | 11.6182 | 5.5575 |
| ENSMUSG00000089824 | Rbm12 | 11.6182 | 17.3439 | 5.2001 |
| ENSMUSG00000051379 | Flrt3 | 12.4303 | 12.6781 | 5.0714 |

**Supplementary Video 1–3:** Representative recordings of spontaneous beating of hiPSC-derived cardiomyocytes under different treatment conditions, demonstrating the effects of EV treatment on beat frequency and contractile behavior.
